# Evidence linking microRNA suppression of essential prosurvival genes with hippocampal cell death after traumatic brain injury

**DOI:** 10.1038/s41598-017-06341-6

**Published:** 2017-07-27

**Authors:** Deborah Kennedy Boone, Harris A. Weisz, Min Bi, Michael T. Falduto, Karen E. O. Torres, Hannah E. Willey, Christina M. Volsko, Anjali M. Kumar, Maria-Adelaide Micci, Douglas S. Dewitt, Donald S. Prough, Helen L. Hellmich

**Affiliations:** 1 Department of Anesthesiology, University of Texas Medical Branch, Galveston, Texas USA; 2 Genus Biosystems, Inc., Northbrook, Illinois USA

## Abstract

The underlying molecular mechanisms of how dysregulated microRNAs (miRNAs) cause neurodegeneration after traumatic brain injury (TBI) remain elusive. Here we analyzed the biological roles of approximately 600 genes - we previously found these dysregulated in dying and surviving rat hippocampal neurons - that are targeted by ten TBI-altered miRNAs. Bioinformatic analysis suggests that neurodegeneration results from a global miRNA-mediated suppression of genes essential for maintaining proteostasis; many are hub genes - involved in RNA processing, cytoskeletal metabolism, intracellular trafficking, cell cycle progression, repair/maintenance, bioenergetics and cell-cell signaling - whose disrupted expression is linked to human disease. Notably, dysregulation of these essential genes would significantly impair synaptic function and functional brain connectivity. In surviving neurons, upregulated miRNA target genes are co-regulated members of prosurvival pathways associated with cellular regeneration, neural plasticity, and development. This study captures the diversity of miRNA-regulated genes that may be essential for cell repair and survival responses after TBI.

## Introduction

To develop targeted therapeutic treatments that will reduce neurodegeneration after traumatic brain injury (TBI), we must delineate which of the hundreds of genes affected by TBI are truly essential for recovery of homeostatic function. The failures in clinical translation of all TBI therapies^1^ indicates that the current consensus on which dysregulated genes and pathways are causally linked to TBI-induced neurodegeneration^2^ needs serious revision. Given that a major confounding factor impeding a full understanding of neurodegenerative signaling is the complexity of TBI-induced mechanisms in hundreds of heterogeneous cell types in the mammalian brain^3^ we leveraged data from our previous microarray study of injury-induced signaling in laser captured populations of dying, Fluoro-Jade-positive (FJ+) and surviving, Fluoro-Jade-negative (FJ−) hippocampal pyramidal neurons^4^ to capture the full diversity of mechanisms of both endogenous neuroprotection and TBI-induced neurodegeneration. Our previous comparative analysis of gene expression in dying and surviving neurons hinted at a coordinated epigenetic regulation, possibly by non-coding microRNAs (miRNAs), of the brain’s cell death and self-repair mechanisms after TBI^4^. MiRNAs negatively regulate gene expression by binding to complementary binding sites in the 3′ untranslated regions and inhibiting translation and/or promoting degradation of target mRNAs^5^. The important role that miRNAs play in coordinating posttranscriptional regulation of protein complexes^6^ and in fine-tuning gene expression by regulating protein expression noise^7^ is highlighted by reports that miRNAs can regulate key physiological processes such as the onset of puberty^8^, aging^9^, brain development^10^, synaptogenesis, and synaptic function^11^. Because dysregulated miRNAs that regulate cell survival genes are implicated in neurodegenerative disorders^12^, linked to increased cell death after brain injury^13^, and in studies of post-mortem human brains, linked to increased neuronal death and decreased neural plasticity in the prefrontal cortices of alcoholics^14^, the therapeutic potential of modulating their expression levels in neurodegenerative disease has been explored^15^. We surmised from our TBI gene expression data (GSE 16735) that although the brain’s endogenous protective responses are evoked in all brain cells regardless of injury status, many prosurvival genes that could mediate this response are markedly suppressed in dying neurons. In the present study, finding that multiple TBI-dysregulated miRNAs are predicted to regulate common dysregulated gene targets in dying and surviving neurons, we make the case for a stressor-threshold model of neurodegeneration^16^ which suggests that TBI-induced neurodegeneration results from low ratios of prosurvival to prodeath genes due, in part, to miRNA-mediated suppression of genes essential for homeostatic brain function.

Here, our analysis shows that virtually all target genes of TBI-dysregulated miRNAs - a subset of the significantly differentially expressed genes previously identified in our studies of TBI-associated gene expression in dying and surviving neurons (GSE 16735)^4 ^- that are differentially suppressed in dying neurons are associated with maintenance of neuronal homeostasis; we infer that suppression of many of these genes would result in disruption of protein homeostasis (proteostasis), the biogenesis, folding, trafficking, and degradation of cellular proteins^17^. Significantly, interrogation of the OMIM (Online Mendelian Inheritance in Man) database shows that dysregulation, mutation, or deletion of many of these genes results in human disease. The global suppression of these essential genes in dying neurons implies profound disturbances in RNA processing and translational machinery, which would impact neural plasticity, brain connectivity, and neuronal survival. On the other hand, we find that upregulated miRNA target genes in surviving hippocampal neurons are co-regulated members of prosurvival pathways associated with cellular regeneration, neural plasticity, and development.

## Results

### Striking differences in expression of miRNA target genes in dying vs surviving neurons

**Figure 1:**
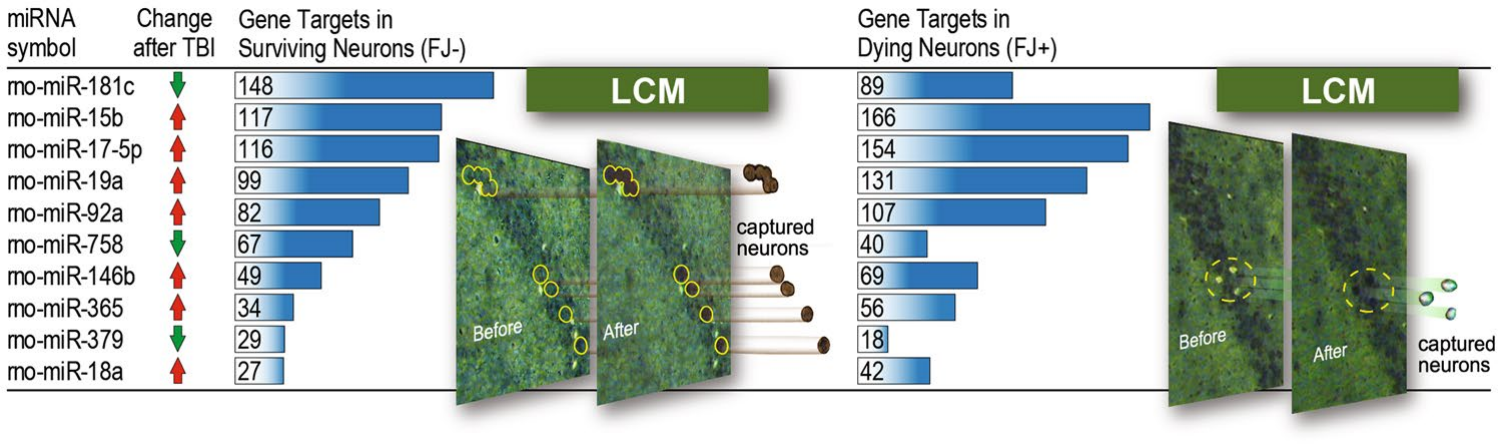
Ten Traumatic brain injury (TBI)-altered microRNAs target approximately 600 pro-survival and/or pro-death genes in dying and surviving hippocampal neurons. In our previous microarray study, we showed that these genes were significantly differentially expressed in dying and surviving neurons 24 h after TBI. Ingenuity pathway analysis miRNA target filter was used to identify predicted gene targets in laser captured dying, Fluoro-Jade-positive (FJ+) and surviving, Fluoro-Jade-negative (FJ−) hippocampal pyramidal neurons.

We first determined expression levels of significantly dysregulated rat miRNAs in uninjured and TBI rat hippocampus using Agilent Rat miRNA microarrays, and confirmed differential expression with individual TaqMan miRNA assays (Supplementary Fig. 1). The experimental parameters (injury level, 24 h time point for molecular analysis, brain region) for miRNA expression profiling were identical to the Rojo study^4^. To identify predicted miRNA targets among the approximately 2000 TBI-dysregulated genes expressed in laser captured dying and surviving neurons identified in our previous study^4^, we used Ingenuity Pathway Analysis miRNA target filter, which provides insights into the biological roles of miRNAs using curated data from experimentally validated miRTarbase and miRecords databases and predicted miRNA-mRNA interactions from TargetScan. We found that ten TBI-dysregulated miRNAs targeted, either singly or frequently in combination, about 600 TBI-dysregulated genes (Fig. 1, manually curated functional data including GeneCard and PubMed links for all miRNA gene targets with gene information shown left of the blue line and miRNA data shown right of the blue line, are provided in Supplementary Tables 1 and 2). Since co-expressed miRNAs have been shown to coordinately regulate canonical cell signaling networks associated with cell death and cell survival^18^, it is notable that we found that all members of the miR-17-92 cluster (miR-17-5p, miR-18a, miR-19a, miR-92a) are upregulated after TBI and these miRNAs co-target and possibly negatively co-regulate many TBI-altered genes. Analysis of the annotated genes that displayed strikingly disparate expression levels in dying and surviving neurons (Table 1, the complete list of differentially expressed miRNA target genes in dying and surviving neurons described in the manuscript are shown in Supplementary Table 3 along with links to OMIM and supporting literature for each gene in Supplementary References) showed that the majority of transcripts are thought to play essential roles in cell function. The biological significance of these genes became clear when we surveyed the literature on their functions to determine the common characteristics of genes associated with neuronal injury and death.

### Upregulated genes reflect an endogenous protective response in dying neurons

Our current analysis reinforces one of the positive findings of our previous study that showed evidence of a cell-autonomous mobilization of neuroprotective genes in dying neurons^4^. Analysis of miRNA gene targets that are upregulated solely in dying neurons shows that these genes have diverse, essential cellular functions: lipid and cholesterol homeostasis (*Abcg4*), glucose, fatty acid, and amino acid metabolism (*Hlcs*), DNA repair (*Nhej1*), cellular metabolism and proliferation of neural stem cells (*Nampt*), and regulation of neuronal migration (*Scrt1*). A few genes such as *Caprin1*, which codes for an RNA-binding protein responsible for mRNA transport to dendrites and neuronal network formation, were upregulated in dying neurons but downregulated in surviving neurons. Genes involved in developmental Wnt signaling and neurite outgrowth (*Ddx3x, Prickle2*) were highly upregulated in both surviving and dying neurons.

### Cell cycle genes suppression is more prominent in dying neurons

Given that cell death induced by DNA damage in postmitotic neurons has been shown to result from aberrant activation and dysregulation of the cell cycle^19^, we noted that several genes involved in regulating the cell cycle (*Heca, Ypel5, Ccpg1*, *Ccng1*) are more downregulated in dying than surviving neurons, the exception being *Mycn* which was more suppressed in surviving neurons. Tumor suppressor genes such as *Jade1* (also known as *Phf17*) that functionally interact with cell cycle regulators are also more suppressed in dying neurons. Other cell cycle regulators (*Haus3, Iars*), including regulators of S phase progression (*Abce1*), G1/S phase transition (*Bzw1*, *Ankrd17*), and cyclin-dependent kinases (*Ccny*), were solely suppressed in dying neurons.

### Preferentially suppressed genes in dying neurons are essential for neuronal function

Three broad categories of genes were preferentially suppressed in dying vs surviving neurons. One group are essential for synaptic function (*Nptx1*), synaptic plasticity (*Nptn*), targeting receptors for degradation (*Grasp1*), vesicle trafficking (*Snx24*), exocytosis (*Anxa7*), modulation of neurotransmitter release (*Cnr1*), regulation of neuronal activity (*Ptk2b*), and neuronal survival and neuroplasticity (*Bdnf*). A second group participates in essential cellular processes, such as fatty acid metabolism (*Acsl4, Far1*), clearance of misfolded proteins (*Jkamp*), regulation of cytoskeleton structure (*Tnfaip1*), regulation of mitochondrial and cytoskeletal dynamics (*Ddhd1, Pak7*), motility (*Actc1*), glycolysis and metabolic reprogramming (*Pfkp*), cell metabolism (*Pgm2l1*), regulation of the mitochondrial permeability transition pore (*Ppm1k*), mRNA stability and post-transcriptional regulation (*Zc3h14*), RNA stabilization (*Hnrnpu*), subcellular protein trafficking (*Srp72, Ap3s1*), degradation of abnormal proteins (*Uba3*), mitotic spindle checkpoint (*Cse1l*), regulation of planar cell polarity (*Fat4*), and regulation of pH, volume, and ionic homeostasis (*Slc9a6*). A third group of genes participate in developmental and regenerative processes: neuronal migration during development (*Astn1*), axon outgrowth and synaptic development (*Cdh10*), regulation of axon regeneration (*Rtn4rl1*), dendritic spinogenesis (*Cttnbp2*), cell growth and survival (*Kras*), neuronal proliferation (*Nap1l2*), neuronal differentiation and migration (*Gpm6a*), cerebellar development (*Mtpn*), neuromuscular junction formation (*App*), neurite outgrowth (*Ccdc64*), neurogenesis (*Grm7*), and regulation of cortical development (*Lrrn3*).

### Genes solely suppressed in dying cells are essential for neuronal homeostasis

The defining characteristic of all the genes that are solely suppressed in dying neurons is that they are essential for maintenance of neuronal function, viability, and homeostasis. One group is involved in synaptic function, development, and plasticity: neurotransmission (*Gabra4, Kcnma1*), homeostatic synaptic scaling (*Epha4*), synaptic vesicle recycling (*Ap2b1, Snap25*), synaptic vesicle exocytosis (*Sv2b*), clathrin-mediated endocytosis (*Aak1*), excitatory neurotransmission (*Grin1*), recycling transmembrane receptors (*Vps35*), trafficking and gating of AMPA receptors (*Cnih2*), glutamate synthesis (*Gls*), regulation of kainite receptors (*Neto2*), neural connectivity and myelination (*Nfasc*), axon guidance (*Epha7*), synapse maturation (*Lrrtm2*), synapse formation (*Nlgn1*), neuronal migration (*Nrn1*, Csde1), neural proliferation (*Ndp*), neurogenesis (*Rps6ka5*), and neuronal plasticity (*Lppr1*).

A second group of genes have roles in multiple cell survival and cytoprotective pathways (*Mapk1, Mapk9, Nr4a3*), antioxidant defenses (*Cat, Lias)*, stress response (*Adcyap1*), regenerative sprouting (*Lppr4*), cell growth (*Tmem209*), and neuroprotection (*Trim2*). Genes such as the heat shock protein co-chaperone *Dnajb9*, which is known to be induced by genotoxic stress, have been shown to protect from apoptotic cell death. This group also includes several relatively uncharacterized genes with hypothesized cytoprotective functions (*Cggbp1,C5orf30*, *Tmcc3*).

A third group of genes participates in diverse homeostatic cell functions: cellular motility and migration (*Ocrl, Fam116a*), polarized cell migration (*Tiam1*), cell adhesion (*Pcdhac2, Rap1a*), cell membrane remodeling (*Chmp1b*), chromatin remodeling (*Chd6*), purine metabolism (*Hprt1*), cholesterol metabolism (*Hmgcr*), lipid biosynthesis and metabolism (*Acsl3*), histone remodeling chaperone (*Asf1a*), metabolism of misfolded proteins (*Cops8*), protein transport (*Rab9a*), cell growth and proliferation (*Gls, Ptpn3, Vrk1, Zfp91*), regulation of ion channels (*Sestd1*), ionic homeostasis (*Cnnm2*), chloride transporter (*Clcn4*), metabolic homeostasis (*Mgea5*), calcium homeostasis (*Stim2*), regulation of actin-filament dynamics (*Cfl2*), metabolism, protein trafficking, signal transduction, apoptosis and cell cycle regulation (*Ywhah*), membrane trafficking (*Rtn3*), nutrient homeostasis (*Rraga*), bioenergetics (*Sdhc*), mitochondrial function (Vdac1), peroxisome biogenesis (*Pex13*), signal transduction (*Sos1*), mechanoreception (*Tmem150c*), actin filament reorganization (*Nckap1*), microtubule dynamics (*Clasp1, Mapre3*), cytoskeletal dynamics (*Elmod1*), mitosis and brain development (*Kif2a,Zfr*), membrane dynamics (*Scamp1*), and assembly of multiprotein complexes (*Dcaf7*).

A fourth group of suppressed genes is essential for transcriptional and translational processes: basal transcription (*Taf9*), DNA repair and maintenance of genome integrity (*Smc5, Usp47*), transcription and nucleotide excision repair (*Gtf2h1*), DNA replication (*Tipin*), RNA metabolism, transport, trafficking (*Hnrnpa3, Hnrnpd*), mRNA splicing (*Sf3b3*), ribosomal RNA processing and export (*Nmd3*), post-transcriptional RNA processing (*Celf2*), miRNA stability (*Papd4*), mitochondrial transcriptional regulation (*Mterfd2*), maintenance of chromatin structure and function (*Hp1bp3*), nucleosome assembly (*Asf1a*), DNA and nucleotide excision repair (*Rad23b, Ercc4*), maintenance of genome stability (*Eepd1*), coordinating the transition between DNA replication and mitosis (*Wee1*), intracellular vesicle trafficking (*Arcn1*), autophagy (*Atg16l1, Map1lc3b*), mitophagy (*Fundc1*), and mitochondrial protein synthesis (*Guf1*).

### Dysregulated miRNA targets have direct links to cell death and disease

Interrogation of the OMIM database showed that many suppressed genes in both dying and surviving neurons are involved in brain development and genetic variations, mutations, or deletions in the majority of these genes are associated with human disease (see OMIM hyperlinks in Supplementary Table 3). Notably the up or downregulation of miRNA target genes in dying or surviving neurons appeared to directly correlate with cell survival or cell death; among the genes that are highly suppressed in surviving neurons are negative regulators of cell survival (*Nlk*), transcriptional repressors (*Trim33*), genes involved in neurodegeneration (*Casp2, Aqp4*), cell death/remodeling processes (*Mmp24*), and induction of inflammatory phenotypes or apoptosis (*Tob1*). Given that over 30% of TBI survivors suffer psychiatric comorbidities^20^, we noted with great interest that genes associated with depression, such as *Slc6a15*, or associated with neuropsychiatric disorders, such as PTSD (*Ncoa1*), are more suppressed in dying neurons. Relatively unknown disease associated genes such as *Clcn4* (X-linked intellectual disability) are among the many that are solely suppressed in dying neurons.

Causal links to cell death pathways and disease also characterize upregulated genes in dying neurons: antiproliferative effects (*Elf1*), negative regulation of cell survival (*Ptpn9*), inflammatory processes (*Ctsc*, *Psmb8*), and apoptosis (*Vps41, Glipr1, Tp53i11*). Several genes are implicated in diseases caused by dysregulated ionic/pH homeostasis (*Slc9a8*), linked to lupus (*Uhrf1bp1*), linked to taupathy (*Mark1*), increased expression linked to Alzheimer’s (*Soat1*), overexpression linked to Huntington’s disease (*Arfgef2*) and linked to mental retardation in Down’s Syndrome (*Sim2*). It is notable that cell-adhesion genes involved in synapse formation (*Nlgn1*) and genes that negatively regulate BACE1 (*Rtn3*) and are markedly suppressed in dying neurons are also implicated in Alzheimer’s pathology.

### Upregulated genes in survival and protective pathways in surviving neurons

Gene expression in surviving neurons clearly correlated with neuronal survival: upregulated genes are involved in developmental processes (*Gfi1b, Prop1*), neuronal development and growth (*Actn2, Marcks*), cell proliferation (*Ghrhr, Ncoa3*), proliferation in neurons and glia (*Kif3b*), notably associated with cell proliferation and survival in cancers (*Cdc37, Itga3, Prickle4, Znf280b*), involved in protective immune response (*Trim14, Nfat5, Ppap2b*), associated with canonical cell survival pathways (*Spock1, Igf1, Notch2, Tyro3*), protect from apoptosis (*Ctf1*), are key regulators of ion channels and synaptic function (*Stom*), calcium homeostasis (*Atp2b2*), ionic homeostasis (*Cftr*), have negative effects on cell death (*Cstb*), act as chaperones to maintain protein structure and function (*Dnajc18*), and are implicated in neuroprotection (*Pgrmc2*).

**Figure 2:**
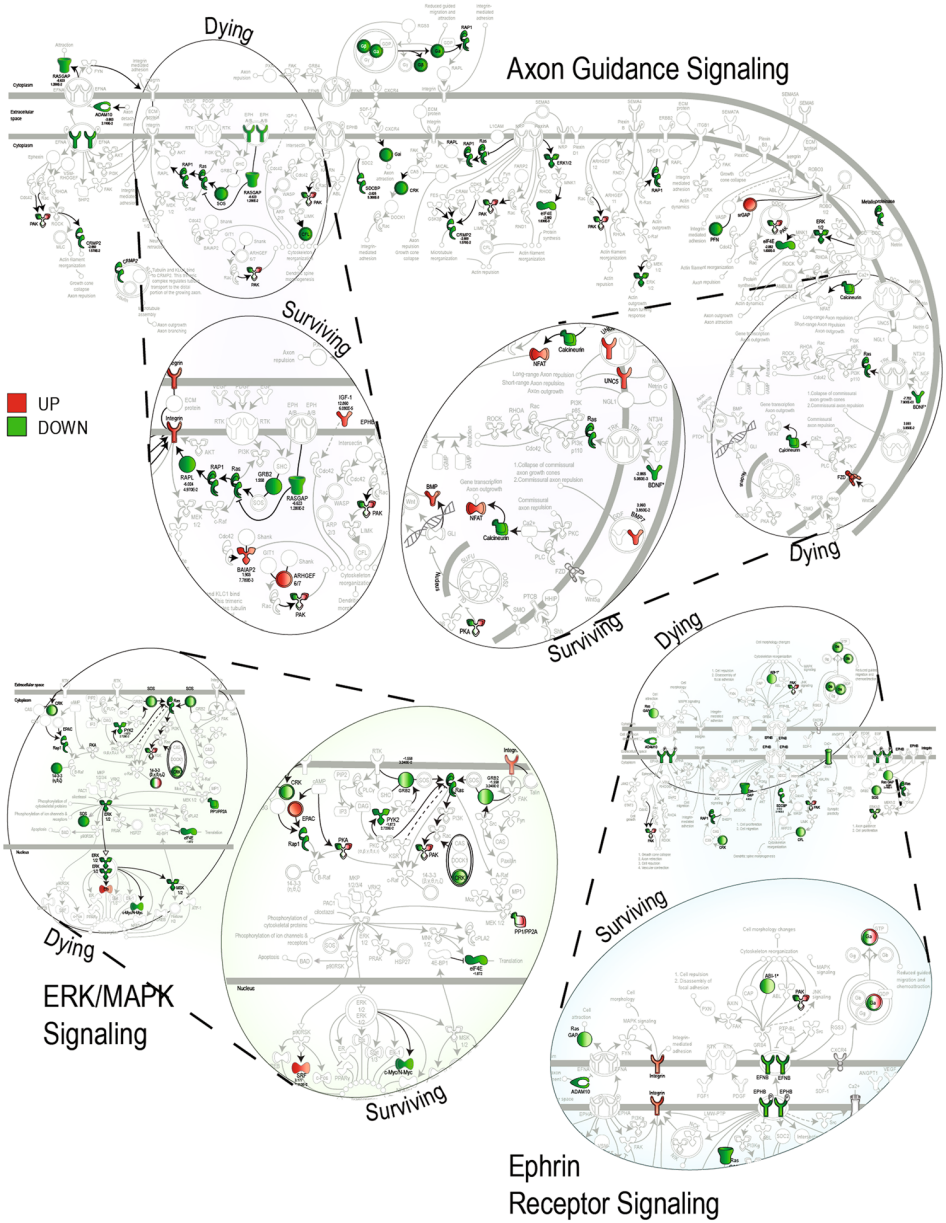
Ingenuity Pathway Analysis of TBI-induced miRNA target genes in dying vs surviving hippocampal neurons. miRNA-targeted genes are disproportionately represented in three prosurvival pathways, axon guidance, ERK/MAPK, and Ephrin receptor signaling. Prosurvival miRNA-target genes that are universally downregulated in the dying neurons are upregulated in surviving neurons (dashed lines connect the same region in dying pathway images with the enlarged surviving pathway images).

The functional relevance of gene expression in surviving neurons can be appreciated by comparing the differences in three prosurvival canonical pathways that were prominently over-represented among the TBI gene lists; differences in the axon guidance, ERK/MAPK, and Ephrin signaling pathways are highlighted by comparing signaling in dying and surviving neurons (Fig. 2A–C, complete pathways are shown enlarged in Supplementary Figs 2–4, gene targets and associated miRNAs are shown in Supplementary Tables 4 and 5). Genes in these pathways, such as *Nfat*, *Bmp*, and *Itga3*, mediate signal transduction pathways linked to cell survival, development, and cell-cell interactions (Supplementary References 259–275).

### High levels of miR-15b in dying neurons negatively correlate with a validated target, *Bdnf*

**Figure 3:**
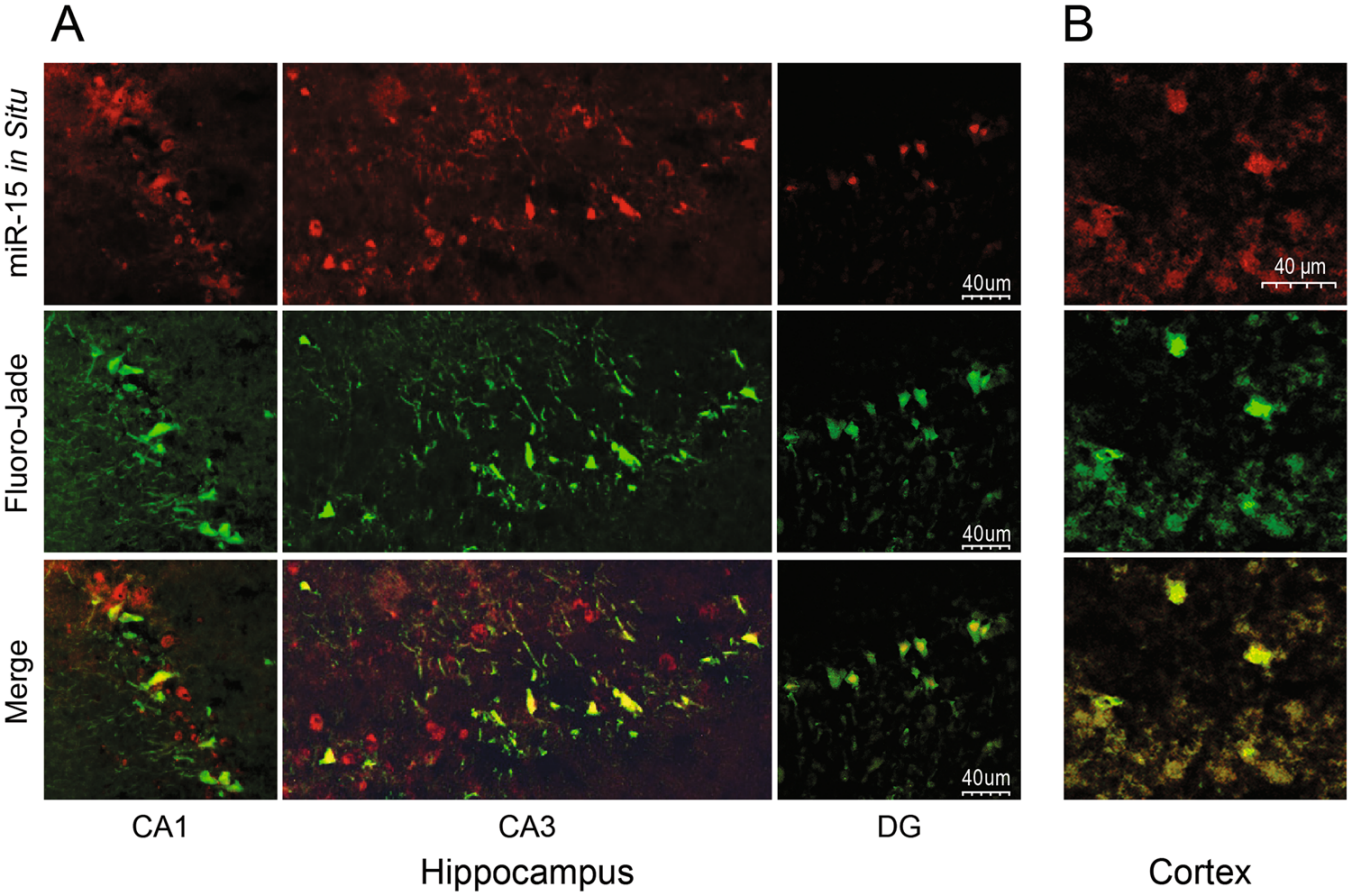
*In situ* hybridization analysis of miR-15b expression using a digoxigen-labeled locked nucleic acid antisense miR-15b probe in frozen sections of the injured rat brain. miR-15b is highly expressed in many but not all dying, Fluoro-Jade-positive (FJ+) neurons in the hippocampal CA1-3 subfields, in all dying (FJ+) neurons of the dentate gyrus (DG) (**A**), and in all FJ+ neurons of the cortex (**B**).

IPAs miRNA target filter showed that in dying neurons miR-15b has the largest number (166) of predicted gene targets among the approximately 2000 TBI-dysregulated genes that were validated by microarray analysis^4^. The deletion of the miR15/16 family is causally linked to chronic lymphocytic leukemia, suggesting that miR-15b has a tumor suppressor/pro-apoptotic function^21^. The high conservation of the miR-15/16 clusters among 44 vertebrate species, including humans and primates, suggests conservation of function^22^ with implications for human TBI. To validate its role in promoting neurodegeneration after TBI, we used *in situ* hybridization (ISH) with a locked nucleic acid (LNA), digoxigenin-labeled antisense probe to determine if miR-15b expression was increased in dying or surviving neurons in TBI brain sections. In comparison to a scrambled miRNA LNA probe (showing only background staining and serving as a negative control probe) and a U6 (small nuclear RNA probe which does not share homology with miRNA sequences available in miRBase) probe that labels all neurons (Supplementary Fig. 5A–C), we found that miR-15b expression is highly upregulated in many dying, FJ+ neurons in all hippocampal subfields (CA1, CA3, dentate gyrus [DG]) and in dying cortical neurons (Fig. 3A,B). Although virtually all FJ+ neurons in the DG and cortex expressed high levels of miR-15b, we found that not all dying, FJ+ neurons in the CA1-CA3 subfields had high levels of miR-15b and conversely, we found miR-15b expressing neurons that were not FJ+; this supports our current hypothesis (see discussion) that gene dosage determines neuronal death, not any specific gene. The miR-15b positive neurons that were FJ− may have had high pre-injury levels of prosurvival genes and therefore, the neurons did not reach the threshold needed to undergo cell death. Conversely, in FJ+ but miR-15b negative neurons, cell death occurs due to the cumulative dysregulation of other miRNAs and genes. We tested the feasibility of using microfluidic qPCR analysis to detect differential expression of miRNAs in laser capture microdissected pools of 30 FJ+ and 30 FJ− neurons (Supplementary Fig. 6A–C). Microfluidic analysis of laser captured neurons has never been reported; in this proof-of-principle experiment, although differences did not reach statistical significance due to the stochastic variability in the six 30 cell pools of dying or surviving neurons, we detected a trend in increased expression of miR-15b and miR-19a in dying neurons.

One of the predicted targets of miR-15b is *Bdnf*, a gene which is essential for cell survival, neuroplasticity, and cognitive functions^23^. *Bdnf* has a large, 2.9 kb 3′UTR with multiple seed binding sites for several miRNAs differentially affected by TBI, miR-15b, miR-146b, miR-17-5p (all increased) and miR-181c (decreased). We showed previously, using microarray analysis of laser captured neurons, that *Bdnf* expression is significantly lower in dying neurons than in adjacent surviving neurons^4^. Since the miR-15b seed binding site is among the most highly conserved (Supplementary Fig. 7A), we used a GeneCopoeia plasmid construct containing the *Bdnf* 3′ UTR and performed dual luciferase reporter assays to find further evidence that miR-15b directly regulates *Bdnf* expression. Although a consistent trend was observed - adding the LNA miR-15b inhibitor to the miR-15b mimic returned luciferase expression levels closer to that of control plasmids in every experiment - the noise and variability in *Bdnf* expression levels prevented the restored expression from reaching statistical significance (Supplementary Fig. 7B). Nevertheless, in aggregate, these data combined with the decreased *Bdnf* levels in laser captured dying neurons (microarray data) and the increased expression of miR-15b in dying neurons (ISH) and increased trends in expression of miR-15b (microfluidic qPCR) support the predicted miR-15b regulation of *Bdnf*.

## Discussion

The extraordinarily diverse functional roles of miRNA gene targets in dying vs surviving neurons appear to defy any logical assignment to categories that inform a mechanistic understanding of neurodegeneration. However, given that every single miRNA gene target in this study was shown in our previous study to be significantly differentially expressed in these neurons after TBI, we found a simple, unifying principle; dysregulation of these genes would profoundly disrupt proteostasis^17^ and consequently neuronal homeostasis after TBI. The biological significance of these results is very clear: in support of previous studies linking disrupted proteostasis to neurodegenerative disease^24, 25^, the gene annotations in the OMIM database show that misregulation, mutation, or deletion of many miRNA gene targets in our study is causally associated with a huge compendium of diverse human diseases, notably neurological and neurodegenerative disorders. For example, mutations in the *Hars* gene are found in patients with inherited peripheral neuropathy with an axonal pathology and genes that regulate tissue-specific alternative splicing, such as *Rbfox1*, are implicated in multiple neurological disorders including autism (see citations for these and all other discussed genes in Supplemental References).

Studies showing that mutations or knockdown of these genes in mice result in deleterious or lethal phenotypes also validate the roles of these miRNA gene targets in neurodegeneration and disease. For example, deletion of *Cacna1c*, which encodes the Cav1.2 subunit of L-type calcium channels, causes increased cell death of hippocampal neurons in knockout mice and mice harboring forebrain-specific conditional knockout of *Cacna1c* display unusually high anxiety-like behavior. Interestingly, polymorphisms in this gene are associated with multiple forms of neuropsychiatric disease that manifest high anxiety in human patients^26^. Mice with mutations in *Grin1* also show abnormal anxiety-like behaviors, a deficiency in fear memory, and a decreased startle amplitude, and mice with *Cfl2* mutations display features of congenital myopathy.

In a recent commentary in Nature about the value of the Exome Aggregation Consortium (ExAC, a database of protein coding sequences from 90,000 plus individuals), Erika Check Hayden discusses how the current views of disease-causing mutations are being drastically revised based on new sequencing data^27^; the exome data clearly show that genetic predisposition is not destiny and in fact, many “fatal” disease-linked mutations exist in otherwise perfectly healthy individuals. Our analysis suggests a plausible explanation for this perplexing observation. There is an obvious and clear gene dosage effect - higher levels of multiple prosurvival genes in surviving vs dying neurons and a greater suppression of essential genes in dying vs surviving neurons - recalling Stanley Korsmeyer’s description of a Bcl-2/Bax rheostat that regulates cell survival or death in cancer cells^28^. Interestingly, we found that multiple miRNAs found downregulated in cancers are upregulated after TBI, which precisely correlates with their roles in regulating genes, such as *Kras*, that are involved in cell survival and proliferation (Supplementary References 276–287). The synergistic, degenerative effects of prosurvival gene suppression in dying neurons are reminiscent of negative epistatic interactions where single mutations are more deleterious in combination than individually^29^. A similar epistatic effect induced by modifier genes may explain variabilities in disease severity in people with familial dysautonomia who carry identical mutations in the IKBKAP gene^30^.

**Figure 4:**
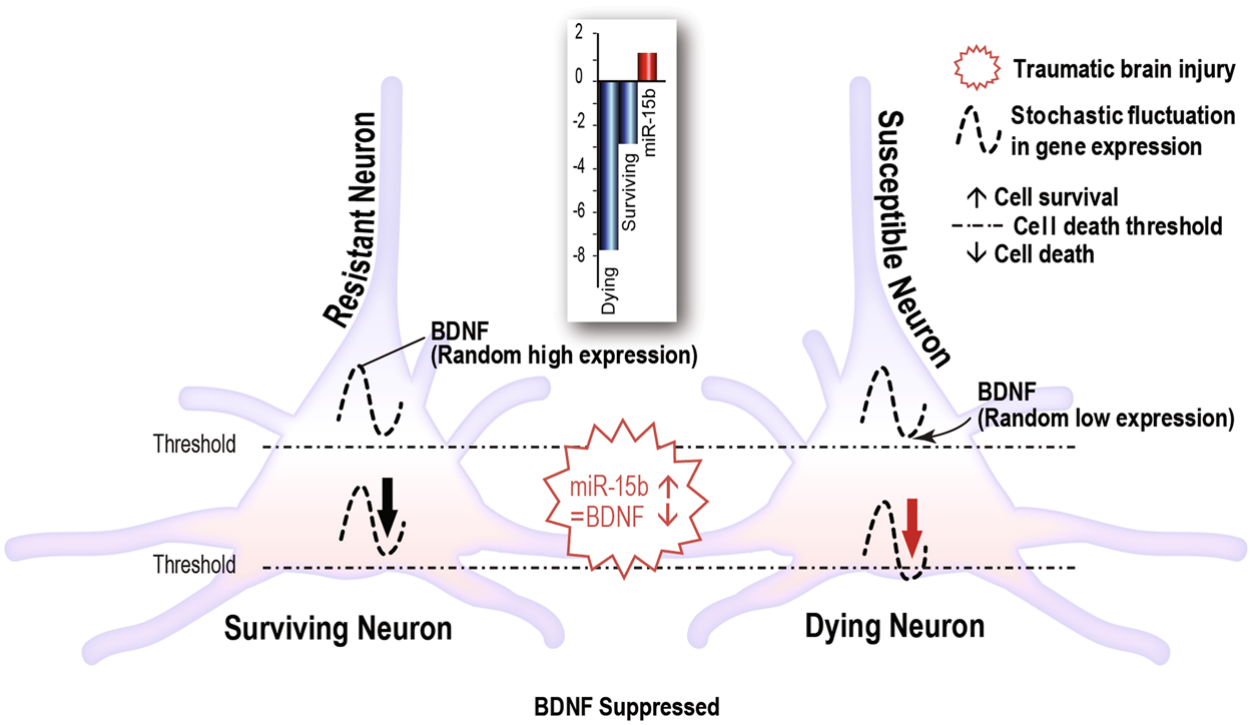
Model of how microRNA regulation of prosurvival target genes could result in differential suppression and differential cell survival after TBI. miR-15b-mediated suppression of random fluctuations in expression levels of pro-survival target genes, such as *Bdnf*, could lower the threshold for cell survival and result in neurodegeneration after TBI. Inset shows expression levels (from separate microarray experiments) of miR-15b in hippocampus of traumatic brain injured (TBI) rats and expression levels of *Bdnf* in pools of laser capture microdissected dying and surviving hippocampal pyramidal neurons after TBI.

Although the basis of selective neuronal vulnerability in neurodegenerative diseases is still unclear, one possible interpretation of our findings is that the differential expression of prosurvival and prodeath genes, which correlate with cell death or cell survival in the hippocampus, supports the stressor-threshold model of neurodegeneration which has been put forth to explain the etiology and sporadic forms of neurodegenerative disease^16^. This model is also supported by the results of our previous study that found stochastic fluctuations in gene expression play a role in determining cell death after TBI^4^. Our analysis suggests a mechanistic model, using *Bdnf* and miR-15b expression levels in dying and surviving cells as an example, for how injury-dysregulated miRNAs could induce differential cell death in adjacent cells that are subjected to the same stressor: because of stochastic fluctuations in *Bdnf* expression in individual neurons, suppression of *Bdnf* expression by TBI-induced miRNAs, such as miR-15b, would push the cell below a critical threshold in some cells and result in cell death (Fig. 4). One limitation of these experiments is that although we tried to measure miRNA expression in small numbers of laser captured neurons using microfluidic qPCR analysis, the noise and variability in the miRNA expression data prevented us from definitive conclusions other than that the trends were in the same direction, i.e. higher expression of miR-15b in dying neurons. One implication of this model is that a therapeutic drug or treatment that reversed the effects of TBI and restored homeostatic levels of one or more dysregulated miRNAs could prevent suppression of some prosurvival gene targets and positively alter the cell survival rheostat. Indeed, in animal models of myocardial infarction, antagomirs to miR-15 reduced infarct size and enhanced cardiac function^31^. Development of miRNA-targeted therapies such as miravirsen, which inhibits the hepatitis C virus by targeting liver-expressed miR-122^32^, give credence to the idea of using drugs targeting TBI-dysregulated miRNAs to improve survival of brain cells.

Many diseases are associated with dysfunction of highly networked signaling hubs^33^, and a comprehensive analysis of the human regulatory network from ENCODE data^34^ showed that miRNAs, acting as key hubs, could shut down entire functional gene units. Given that many of the miRNA-targeted genes are themselves hub genes and transcriptional regulators (Supplementary References) and given that many of the rat genes suppressed in dying neurons have counterparts that been shown to be essential in the human genome^35^, this suggests that injury-altered miRNAs could coordinately induce a wide-spread dysregulation of critical cell survival pathways in the injured brain. Moreover, since we know that the synchronous activity in brain networks correlates with expression of dozens of genes involved in activity of ion channels and synaptic function^36^ (such as the ones in our study), and we know that brain injury causes massive disruptions in brain connectivity and the brain default network^37, 38^ and that disturbances in brain connectivity cause many neurological diseases^39^, then the large numbers of suppressed genes associated with synaptic function would predict a broad disruption of functional connectivity and cognitive function.

These data also suggest a molecular mechanism explaining why TBI is a risk factor for age-related and comorbid neurodegenerative and neuropsychiatric disorders^40^. *Bdnf* and *Fgf2* are among multiple miRNA target genes linked to several psychiatric disorders including depression^41^. Moreover, potassium channel genes, such as *Kcnma1*, which have been implicated in METH and alcohol addiction and genes, such as *Eif2s1* and *Ehd4*, are implicated in drug abuse or reward circuits (Supplemental References). Disruption or dysregulation of synaptic function genes such as *Scn1a* - loss of function is associated with genetic epileptic disorders^42^ and *Sv2b*, is associated with TBI comorbidities such as epilepsy^43^. It is notable that the only drug that has proved neuroprotective in multiple TBI animal models is the antiepileptic drug, levetiracetam^44^. Finally, a recent study of the aging rat brain found that age-related down-regulation of protein-coding genes is inversely correlated with a genome-wide up-regulation of non-coding genes, including miRNAs^45^. Until now, a plausible mechanism for how TBI accelerates aging and poses a risk factor for Alzheimer’s disease^46^ has been obscure; our study suggests that TBI-induced miRNAs dysregulate multiple genes linked to AD and genes such as *Zmpste24*, a gene causally linked to the premature aging disease progeria.

In summary, we have captured the full diversity of TBI-induced neurodegenerative mechanisms by focusing on miRNA gene targets that are differentially expressed in pure populations of dying and surviving neurons. We do want to stress that this captures only a moment in time, 24 h after TBI, and all these observations pertain only to that time point. If we could follow these surviving neurons *in vivo*, it is likely that gene expression changes in some of the “surviving” neurons will push these cells below a critical threshold necessary for survival and they may die at a later post-injury timepoint. The translational implication of our study is summed up in a recent review of the polygenic nature of many psychiatric disorders; the finding that individual genetic differences are common to multiple disorders implies not only shared pathophysiology but unexpected opportunities to broaden the use of single disease-targeted therapeutics for other disorders^47^. That is, miRNA-targeted therapeutics for TBI may also prove beneficial for other neurodegenerative disorders.

## Methods

### Animal surgery

All animal experiments were approved by the Institutional Animal Care and Use Committee of the University of Texas Medical Branch, Galveston, Texas and conducted in accordance with the guidelines and regulations in the National Institutes of Health Guide for the Care and Use of Laboratory Animals (8^th^ edition, National Research Council). Lateral fluid percussion traumatic brain injury (TBI) was performed on 300–400 g male Sprague-Dawley rats as previously described^4^.

### Agilent microRNA microarray and bioinformatic analysis

Total RNA, including miRNA, was isolated from rat hippocampi (n = 3 each for naïve control, and TBI) at GenUs Biosystems and Agilent Rat miRNA arrays (Agilent Technologies, Santa Clara, CA), consisting of 938 probes that target 350 unique miRNAs, were performed according to manufacturer’s protocols. Microarrays were scanned, and data was uploaded into GeneSpring GX11 (Agilent Technologies), normalized, and analyzed as described in Rojo *et al*.^4^.

Differentially expressed genes >1.5-fold and T-test p-value < 0.05 in dying and surviving neurons relative to sham controls and differentially expressed miRNAs >1.2-fold, T-test p-value < 0.05 in TBI vs. naive whole hippocampus were uploaded into Ingenuity Pathway Analysis (IPA). IPA’s miRNA Target Filter was used to match differentially expressed miRNAs to their predicted targets differentially expressed in the opposite direction. The miRNA Target Filter is based on experimentally validated microRNA-mRNA target interactions from miRecords, plus thousands of additional miRNA-mRNA interactions manually curated from additional peer-reviewed literature. The target filter identified the same 10 miRNAs whose sequence target 613 differentially expressed genes in dying (Fluoro-Jade-positive) neurons vs control (uninjured neurons in sham-operated rat brains), 567 differentially expressed genes in surviving (Fluoro-Jade-negative) vs control neurons, and 1040 differentially expressed genes when both injured and uninjured neurons are compared to sham controls. A Canonical Pathway analysis in IPA identified ERK/MAPK Signaling, Ephrin Receptor Signaling, and Axonal Guidance Signaling as the three pathways with the most significant enrichment (p < 0.05) of differentially expressed genes targeted by one of the 10 miRNAs (shown in Supplementary Tables 4 and 5). To understand the biological roles of each miRNA target gene in dying and surviving neurons, we manually curated functional data using GeneCards, a database of human genes (automatically curated and integrated data from approximately 125 web-based sources) that provides genomic, proteomic, transcriptomic, genetic, clinical, and functional information on all known and predicted human genes and interrogating the published literature on each gene. For most genes, a PubMed hyperlink to a relevant publication is provided in addition to an active hyperlink to GeneCards.

### Whole hippocampal tissue RNA isolation and qPCR to confirm microRNA microarray data

Total RNA, including miRNA, was isolated from rat hippocampal tissue (6 naïve control, 6 TBI) using the mirVana isolation kit (Ambion, Austin, TX P/N AM1560) following manufacturer’s protocols. RNA was then DNase-treated using DNA-free (Ambion, P/N AM1906) for 20 min at 37°C. One µl of total RNA from each sample was assayed on an Agilent 2100 Bioanalyzer (Agilent Technologies, Santa Clara CA) to assess the quality and determine quantity. Ten ng of total RNA was reverse transcribed using the Taqman microRNA reverse transcription kit (P/N4366596, Applied Biosystems by Life Technologies, Carlsbad, CA) according to the manufacturer’s protocol. Real-Time PCR was performed on the MX3000 P thermal cycler (Stratagene, La Jolla, CA). Taqman microRNA assays to specific miRNA’s were purchased from (Applied Biosystems by Life Technologies, Carlsbad, CA. P/N 4427975). U6 was used as the endogenous control to normalize miRNA data. All data collected was analyzed using the MXPro software (Stratagene, now Agilent), a data analysis tool for sample comparison using the ΔΔCT method for calculating the relative quantitation of miRNA expression.

### Sectioning of rat brains for ISH and Fluoro-Jade C analysis

At 24 h post-FPI, rat brains (n = 12) were dissected out; immediately frozen on dry ice and stored −80°C. For cryosectioning, brains were removed from the freezer, placed in the cryostat until they were thawed to approximately −20°C. Brains were sectioned until the specified hippocampal region (Bregma −3.15 mm to Bregma −4.15 mm) was reached and 10 μm coronal serial sections were collected and mounted on uncoated, precleaned Superfrost glass slides (Fisher Scientific, Pittsburgh, PA). Slides were kept at −20°C until sectioning was completed.

### *In situ* hybridization for miR-15b

Ten µm fresh frozen coronal sections were fixed in 10% formalin overnight at ambient room temperature. Slides were rinsed in 1X PBS three times; 3 min each before Proteinase-K treatment. Slides were placed into a humid chamber and 300 uL of a 2 ug/mL concentration of Proteinase K solution (stock—20 mg/mL) was pipetted onto each section and covered with a strip of Parafilm. Slides were incubated at 37°C in a hybridization oven for 10 min. Slides were rinsed in 1X PBS two times; 5 min. Slides were then dehydrated in a series of ethanol washes (immersed 10 times and then submerged for 1 min in the following concentrations—70%, 96%, and 99.9%, respectively). Sections were then allowed to dry for 15 min on a Kimwipe. In preparation for the hybridization of the double-DIG LNA probe to miR-15b, hybridization buffer was diluted 1:1 in RNase free water. MiR-15b probe was denatured at 90°C for 4 min. Denatured probes were diluted with hybridization buffer to a concentration of (1 nM to 80 nM) depending on the miRNA. Then, 50 µL of diluted probe was added to each section and covered with Parafilm. Slides were placed in a humidity chamber and transferred into a hybridization oven set at 51°C for 1 h. Slides were then placed in Coplin jars and washed in room temperature 5X SSC for 5 min. Subsequent 5 min washes in preheated 51°C SSC placed in the hybridization occurred in the following order: 5X, 1X, 1X, 0.2X, 0.2X. A final wash at ambient room temperature in 0.2X SSC was completed before the slides were transferred to another Coplin jar with 1X PBS. A hydrophobic barrier was drawn with a delimiting pen (DAKO) to keep staining reagents localized on the tissue. Sections were blocked in 5% normal goat serum +0.3% triton X-100 for 30 min at room temperature to reduce the overall non-specific binding of the primary antibody. Next, 100 uL of primary antibody (Dylight 594 anti-DIG 1:20 concentration, Vector Biolabs #DI-7594) +1% goat serum in PBS incubated on the slides overnight at 4°C inside the humidity chamber. The following day slides were removed and washed in 1X PBS three times; 10 min each time. Sections were then rinsed in distilled MilliQ water and incubated in a 0.06% potassium permanganate solution for 1 min to reduce background artifacts from subsequent fluorescent staining. Slides were then washed for 2 min in distilled water. Sections were then stained with FluoroJade-C (FJ-C) a neuronal marker of cell death shown previously to preferentially bind to dying, degenerating cells. FJ-C was diluted to 0.0001% in distilled water with 0.1% acetic acid and the sections were incubated for 7 min. Following FJ-C staining the slides are washed in distilled water three times, 1 min for each wash. Slides were mounted with media composed of 80% glycerol and 0.1% acetic acid. An Olympus BX51 Fluorescent Microscope was used to visualize hippocampal and surrounding cortical regions.

### Staining for laser capture microdissection

Frozen sections immediately adjacent to miR-15b ISH/FJC labeled slides were removed from the cryostat and placed at ambient temperature for approximately 30 sec then promptly fixed for 1 min in 75% ethanol; rinsed in RNase-free water (1 min); stained with 1% cresyl violet (20 sec); rinsed in RNase-free water (30 sec × 2); stained with. 001% Fluoro-Jade C (4 min); rinsed in RNase-free water (1 min × 3); dehydrated in 95% ethanol (30 sec) then 100% ethanol (30 sec); rinsed in xylene (2.5 min × 2); and allowed to air-dry for 10–15 min in a chemical fume hood. Solutions were prepared under RNase-free conditions and the cresyl violet and Fluoro-Jade C solutions were filtered (0.2 micron filter) before use. We have adapted conventional Fluoro-Jade C staining protocols to achieve maximum preservation of RNA integrity throughout the laser capture microdissection procedures.

### Laser Capture Microdissection

Laser capture microdissection (LCM) was performed using a PixCell IIe laser capture microscope with an infrared diode laser (Life Technologies, CA). Using serial sections immediately adjacent (before and after) to the miR-15b ISH/FJC labeled slides, we identified dying, Fluoro-Jade positive neurons and adjacent surviving, Fluoro-Jade negative neurons and laser captured them from the ipsilateral (directly under the injury site) rat hippocampus. Contamination from adjacent cells was minimized by using the smallest laser spot size (7.5 micron) and a power setting range of 75–100 mW with pulse duration of 0.45–0.85 ms, the last two settings adjusted as necessary for optimum capture. Surviving and dying neurons were separately captured on thermoplastic films of CapSure Macro LCM Caps (Life Technologies, CA). Caps were then secured on 0.5 mL tubes with 100 μL lysis solution from the RNAqueous-MicroRNA isolation kit (Ambion, Austin, TX) and vortexed for 15 sec, stored at −20°C, and vortexed 30 sec before the RNA isolation procedure.

### MicroRNA Real-Time PCR for LCM samples using Fluidigm’s dynamic array IFCs

Thirty dying (n = 6 pools) and 30 adjacent surviving (n = 6 pools) neurons in the CA1-CA3 region of the hippocampus were collected by LCM. Cells were lysed with buffer obtained from the RNAqueous Micro Kit (Life Technologies). Total RNA was isolated using the RNAqueous Micro Kit according to the manufacturer’s protocols. Total RNA was eluted in 20 µl of nuclease free water and Dnase treated at 37°C for 20 min. Total RNA was assayed on an Agilent 2100 Biolanalyzer (Agilent Technologies) to determine the quantity and integrity of the RNA samples. RIN (RNA integrity numbers) ranged from 6.3–6.8. Reverse Transcription was performed using the RT primers provided in the TaqMan MegaPlex Primer Pool A and the MicroRNA Reverse Transcription Kit. The resulting cDNA was then preamplified using the pooled Preamp primers provided in the MegaPlex kit for 15–18 cycles. The preamped products were then diluted 1:10. Forty cycles of RT-PCR were performed, using the microfluidic 48.48 Dynamic Array IFCs. The data was then analyzed by Fluidigm scientists using Fluidigm’s Real-Time Data Analysis Software. Since the data from single cells or limited numbers of cells show great stochastic variation in mRNA amounts that routinely ranges from 10–1000 fold, Fluidigm has developed statistical analysis tools and software specifically suited for analysis of limited cells. Microfluidic qPCR data was analyzed with SINGuLAR™ Analysis Toolset 2.1 which enables one-way ANOVA on single-cell data.

### Dual Luciferase Reporter Assays

HEK293 cells were plated in 96 well plates (3.0–4.0 × 10^4^) in antibiotic-free complete growth media (EMEM + 10% FBS) 24 h prior to transfection. Transfections (three separate experiments) were performed in triplicate by adding 10 ul of transfection complex containing 0.5 ul Lipofectamine 2000 (Invitrogen), 100 ng of *Bdnf* miRNA Target Sequence 3′UTR expression clone (GeneCopoeia), 60 nmol/L miR-15b mimic (Dharmacon), or 37 nmol/L antisense LNA oligo (Exiqon). Experimental conditions included plasmid alone, plasmid + miR-15b mimic, and plasmid + miR-15b mimic and LNA inhibitor. Wells with cells alone were used to subtract background expression when read on the plate reader. Then, 24 h after transfection cells, were lysed and both Firefly luciferase and Renilla luciferase expression was measured using the Luc-Pair miR Luciferase Assay (GeneCopoeia) in a GloMax^®^-Multi+ Detection System with Instinct™ Software (Promega). After background subtraction, Firefly luminescence was normalized to Renilla luminescence producing relative luciferase unit (RLU) values. Data are reported as a percent change from the BDNF plasmid alone levels, displayed with 95% confidence intervals. A paired t-test was used to compare the miR-15b mimic groups and miR-15b mimic plus inhibitor groups to BDNF plasmid alone groups. All calculations were done in R.

### Data Availability.

The gene and microRNA expression data reported in this paper have been uploaded to NCBI’s Gene Expression Omnibus (GEO) under accession numbers GSE16735, GSE59646.

## Electronic supplementary material

Supplementary figures and references

Table S1

Table S2

Table S3

Table S4

Table S5
