# Publisher Correction: Evidence linking microRNA suppression of essential prosurvival genes with hippocampal cell death after traumatic brain injury

**DOI:** 10.1038/s41598-018-31991-5

**Published:** 2018-10-08

**Authors:** Deborah Kennedy Boone, Harris A. Weisz, Min Bi, Michael T. Falduto, Karen E. O. Torres, Hannah E. Willey, Christina M. Volsko, Anjali M. Kumar, Maria-Adelaide Micci, Douglas S. Dewitt, Donald S. Prough, Helen L. Hellmich

**Affiliations:** 1 Department of Anesthesiology, University of Texas Medical Branch, Galveston, Texas USA; 2 Genus Biosystems, Inc., Northbrook, Illinois USA

Correction to: *Scientific Reports* 10.1038/s41598-017-06341-6, published online 27 July 2017

In this Article, Figure 1B is omitted. The correct Figure 1 appears below.

As a result, the Figure legend,

“Ten Traumatic brain injury (TBI)-altered microRNAs target approximately 600 pro-survival and/or pro-death genes in dying and surviving hippocampal neurons. In our previous microarray study, we showed that these genes were significantly differentially expressed in dying and surviving neurons 24 h after TBI. Ingenuity pathway analysis miRNA target filter was used to identify predicted gene targets in laser captured dying, Fluoro-Jade-positive (FJ+) and surviving, Fluoro-Jade-negative (FJ−) hippocampal pyramidal neurons”.

**Figure 1:**
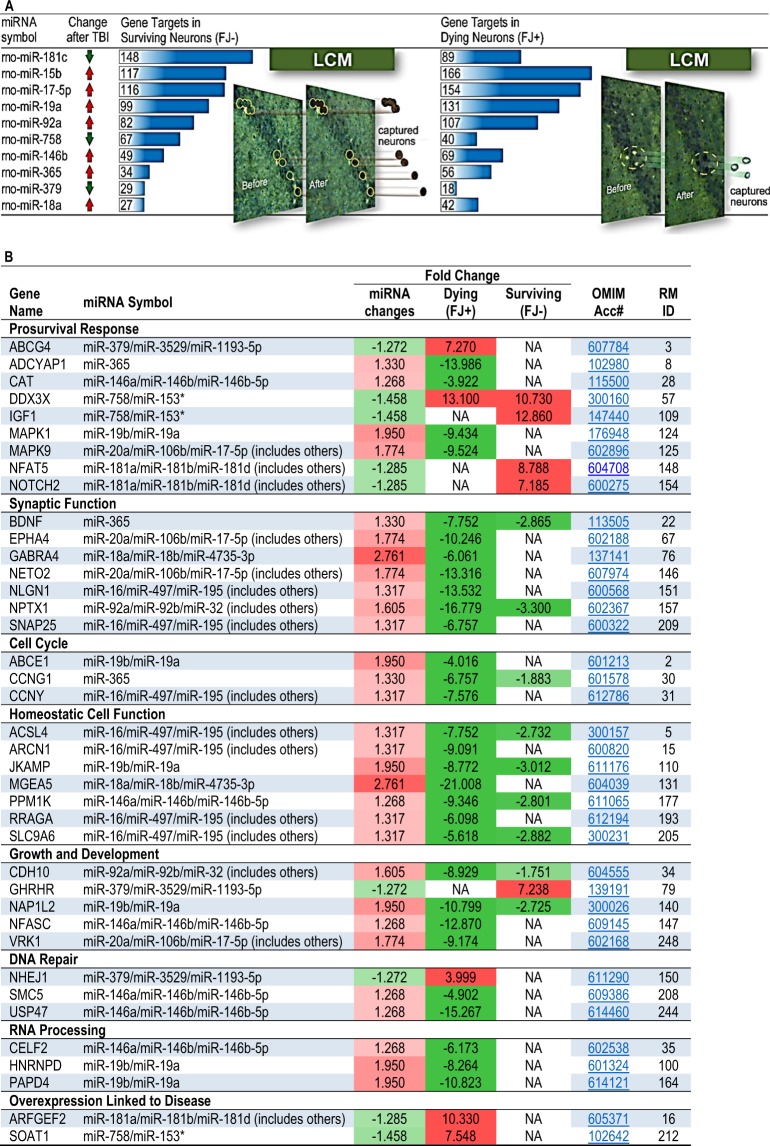
Ten Traumatic brain injury (TBI)-altered microRNAs target approximately 600 pro-survival and/or pro-death genes in dying and surviving hippocampal pyramidal neurons that were obtained by laser capture microdissection 24 h after TBI. (**A**) Ingenuity pathway analysis miRNA target filter was used to identify predicted gene targets in dying, Fluoro-Jade-positive (FJ+) and surviving, Fluoro-Jade-negative (FJ−) neurons. These genes were identified in our previous microarray study as significantly differentially expressed in dying and surviving neurons. (**B**) Differentially expressed miRNA target genes in dying and surviving neurons play essential roles in neuronal function and their misregulation is associated with human disease (see Online Mendelian Inheritance in Man database). The complete, annotated list of differentially expressed miRNA target genes in dying and surviving neurons is shown in Supplementary Table 3.

should read:

“Ten Traumatic brain injury (TBI)-altered microRNAs target approximately 600 pro-survival and/or pro-death genes in dying and surviving hippocampal pyramidal neurons that were obtained by laser capture microdissection 24 h after TBI. (A) Ingenuity pathway analysis miRNA target filter was used to identify predicted gene targets in dying, Fluoro-Jade-positive (FJ+) and surviving, Fluoro-Jade-negative (FJ−) neurons. These genes were identified in our previous microarray study as significantly differentially expressed in dying and surviving neurons. (B) Differentially expressed miRNA target genes in dying and surviving neurons play essential roles in neuronal function and their misregulation is associated with human disease (see Online Mendelian Inheritance in Man database). The complete, annotated list of differentially expressed miRNA target genes in dying and surviving neurons is shown in Supplementary Table 3”.

Additionally, there are typographical errors in the text. In the Results section:

“We found that ten TBI-dysregulated miRNAs targeted, either singly or frequently in combination, about 600 TBI-dysregulated genes (Fig. 1, manually curated functional data including GeneCard and PubMed links for all miRNA gene targets with gene information shown left of the blue line and miRNA data shown right of the blue line, are provided in Supplementary Tables 1 and 2)”.

should read:

“We found that ten TBI-dysregulated miRNAs targeted, either singly or frequently in combination, about 600 TBI-dysregulated genes (Fig. 1A, manually curated functional data including GeneCard and PubMed links for all miRNA gene targets with gene information shown left of the blue line and miRNA data shown right of the blue line, are provided in Supplementary Tables 1 and 2)”.

In the same section:

“Analysis of the annotated genes that displayed strikingly disparate expression levels in dying and surviving neurons (Table 1, the complete list of differentially expressed miRNA target genes in dying and surviving neurons described in the manuscript are shown in Supplementary Table 3 along with links to OMIM and supporting literature for each gene in Supplementary References) showed that the majority of transcripts are thought to play essential roles in cell function”.

should read:

“Analysis of the annotated genes that displayed strikingly disparate expression levels in dying and surviving neurons (Fig. 1B, the complete list of differentially expressed miRNA target genes in dying and surviving neurons described in the manuscript are shown in Supplementary Table 3 along with links to OMIM and supporting literature for each gene in Supplementary References) showed that the majority of transcripts are thought to play essential roles in cell function”.

