# Supplementary material for: Evidence linking microRNA suppression of essential prosurvival genes with hippocampal cell death after traumatic brain injury
Source: Sci Rep. 2017 Jul 27;7:6645. doi: 10.1038/s41598-017-06341-6 (PMC5532254; doi:10.1038/s41598-017-06341-6)
Supplement: Supplementary file 1 — Supplementary figures and references [file 41598_2017_6341_MOESM1_ESM.pdf]

Evidence linking microRNA suppression of essential prosurvival genes with hippocampal cell death after traumatic brain injury

Deborah Kennedy Boone[1], Harris A. Weisz[1], Min Bi[1],
Michael T. Falduto[2], Karen E. O. Torres[2], Hannah E. Willey[1],
Christina M. Volsko[1], Anjali M. Kumar[1], Maria-Adelaide Micci[1],
Douglas S. Dewitt[1], Donald S. Prough[1] and Helen L. Hellmich*[1]

[1] Department of Anesthesiology, University of Texas Medical Branch, Galveston, Texas

[2] Genus Biosystems, Inc. Northbrook, IL

* Corresponding author email: hhellmic@utmb.edu

Supplementary Figure 1.

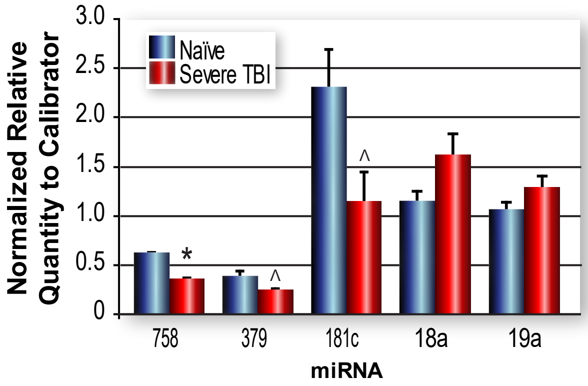

Supplementary Figure 1. Confirmation of trends in whole hippocampus microRNA microarray expression data using individual TaqMan microRNA assays. Changes in all three downregulated miRNAs (miR-181c, miR-379, miR-758) and two upregulated miRNAs (miR-181 and miR-19a) in TBI hippocampus were confirmed. Increased expression of miR-15b was independently validated by in situ hybridization (see Fig. 3 in manuscript). Differences in expression of remaining miRNAs could not be confirmed by qPCR. Total RNA and miRNA was isolated from (naïve control or TBI rat hippocampus (n=3/group) using the mirVana isolation kit (Ambion, Austin, TX). RNA was DNase-treated (Ambion). 1 ng of total RNA was assayed on an Agilent 2100 Biolanalyzer (Agilent Technologies, Santa Clara, CA) to verify each sample. Total RNA (10 ng) was reverse transcribed using the Taqman microRNA reverse transcription kit (Applied Biosystems, Life Technologies, Carlsbad, CA). Real-Time PCR was performed on an MX3000P (Stratagene, La Jolla, CA). Taqman microRNA assays were used for specific miRNA's (Applied Biosystems) with U6 as the endogenous control normalized to each miRNA. Data was analyzed with the MXPro software (Stratagene) tool using the ΔΔCT method. Students t distribution (two-tailed t-test) compared Naïve vs. Severe TBI, *P<0.05 vs. Naïve, ^ P= 0.07 vs Naive.

Supplementary Figure 2.

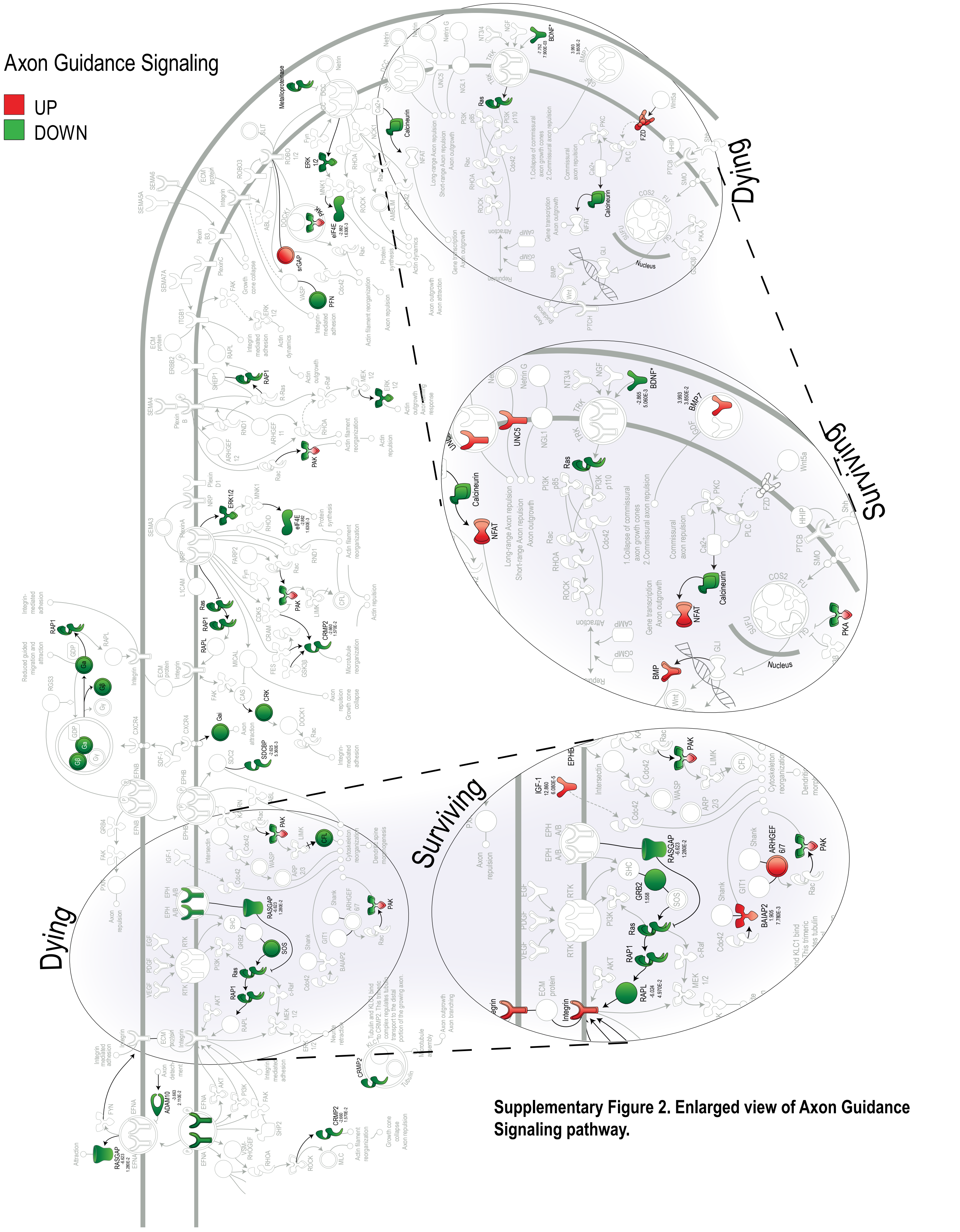

Supplementary Figure 2. Enlarged view of Axon Guidance Signaling pathway.

Supplementary Figure 3.

ERK/MAPK Signaling

UP

DOWN

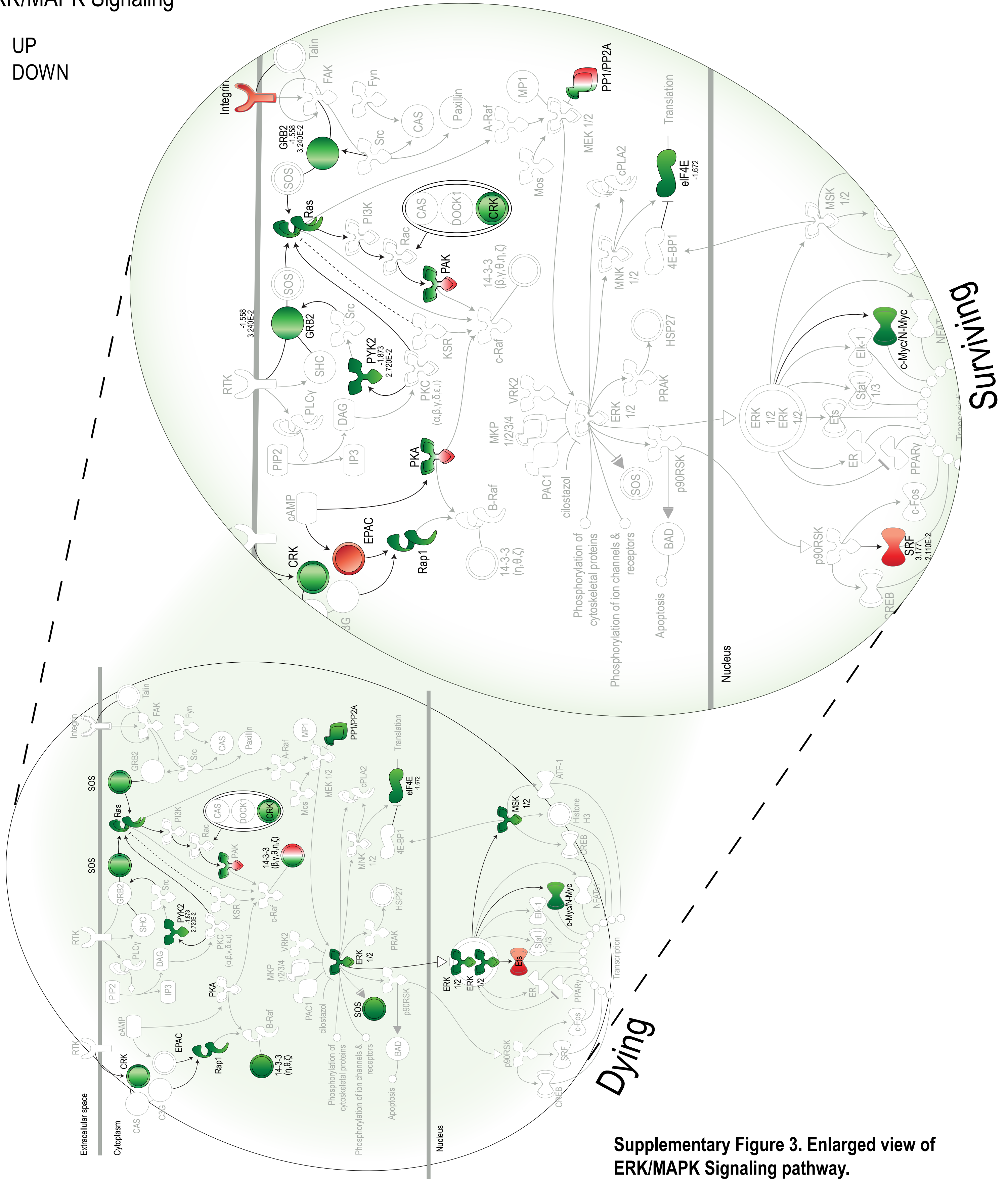

Supplementary Figure 3. Enlarged view of ERK/MAPK Signaling pathway.

Supplementary Figure 4.

Ephrin Receptor Signaling

UP

DOWN

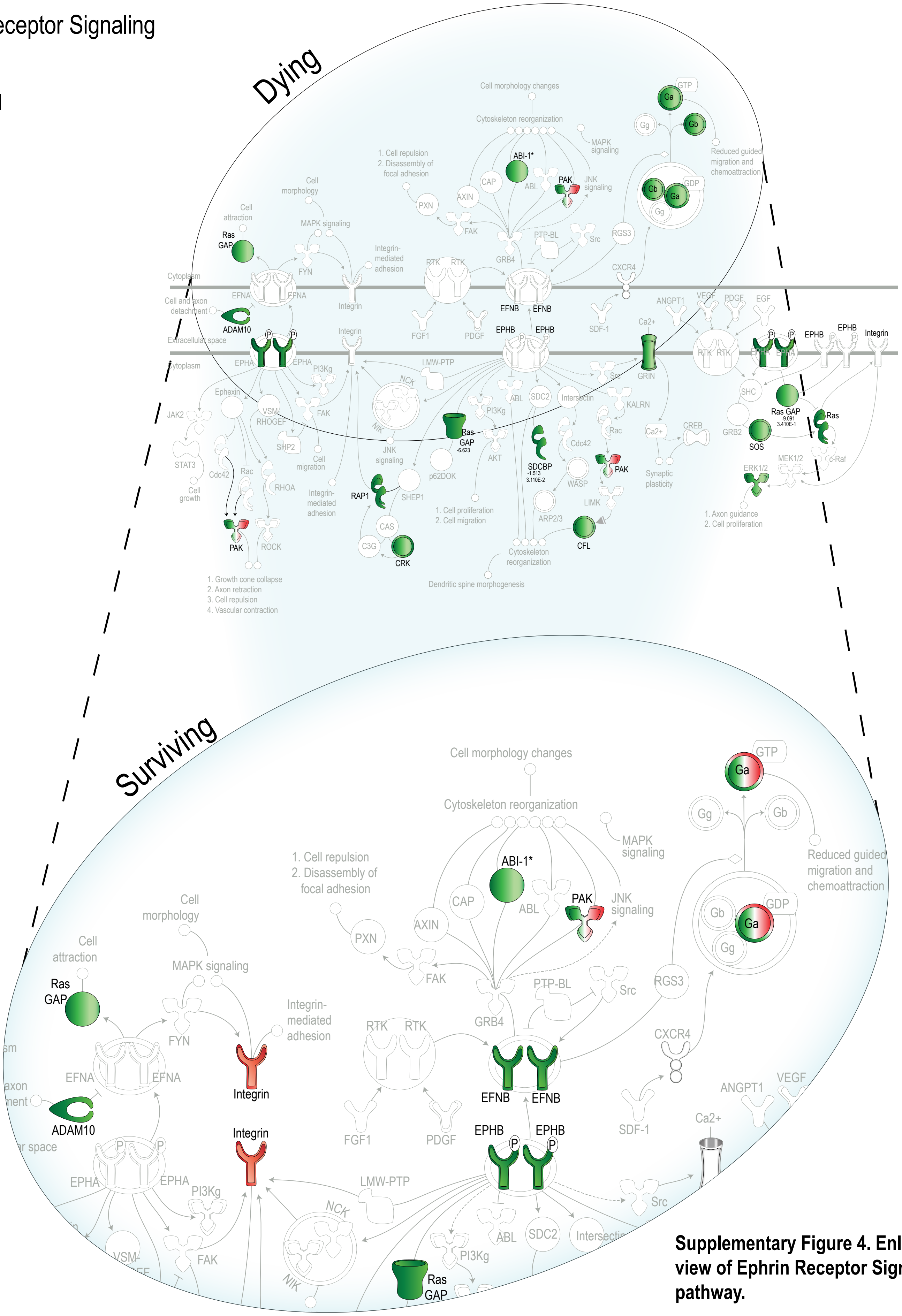

Supplementary Figure 4. Enlarged view of Ephrin Receptor Signaling pathway.

Supplementary Figure 5.

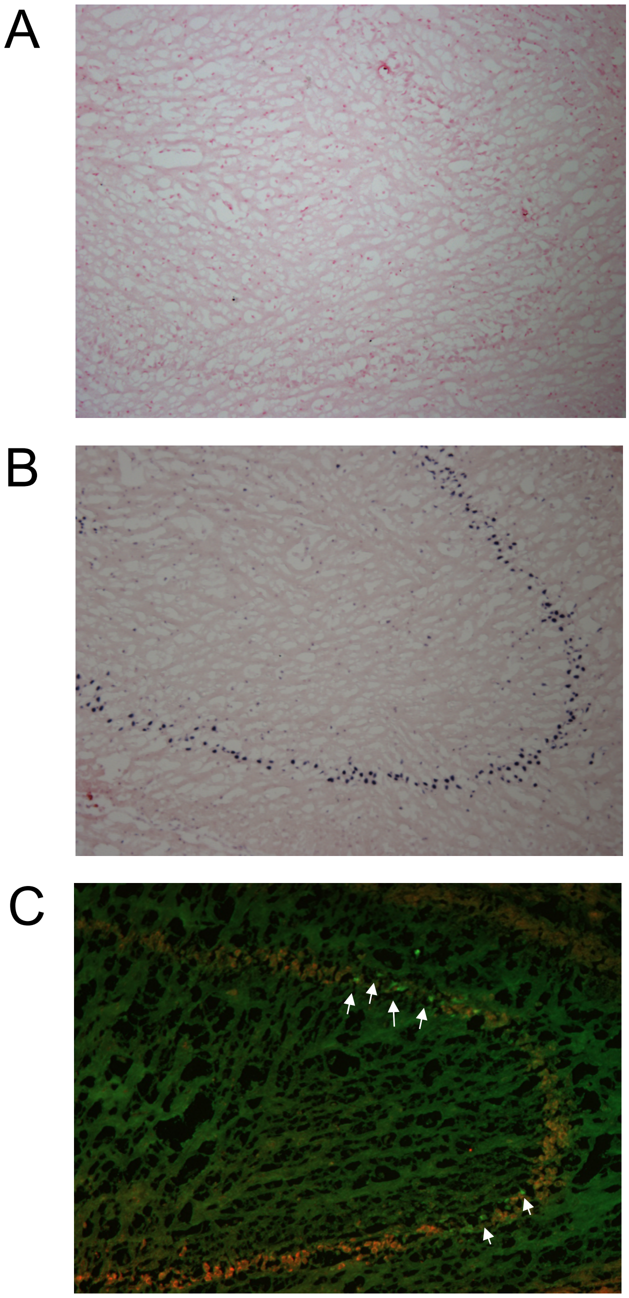

Supplementary Figure 5. In situ hybridization with locked nucleic acid scrambled and U6 probes in the hippocampal CA1, 2, and 3 pyramidal layers (A-C). In situ labeling with the scrambled probe shows non-specific binding, 10X magnification (A), anti-Digoxigenin-labeled U6 probe shows widespread labeling of pyramidal layer neurons, 10X magnification (B), and co-labeling with anti-DIG antibody to DIG-labeled U6 (red) and Fluoro Jade C (green) which is specific for dying neurons (white arrows) shows typical patterns of neurodegeneration in brain injured rats, 10X magnification (C).

Supplementary Figure 6.

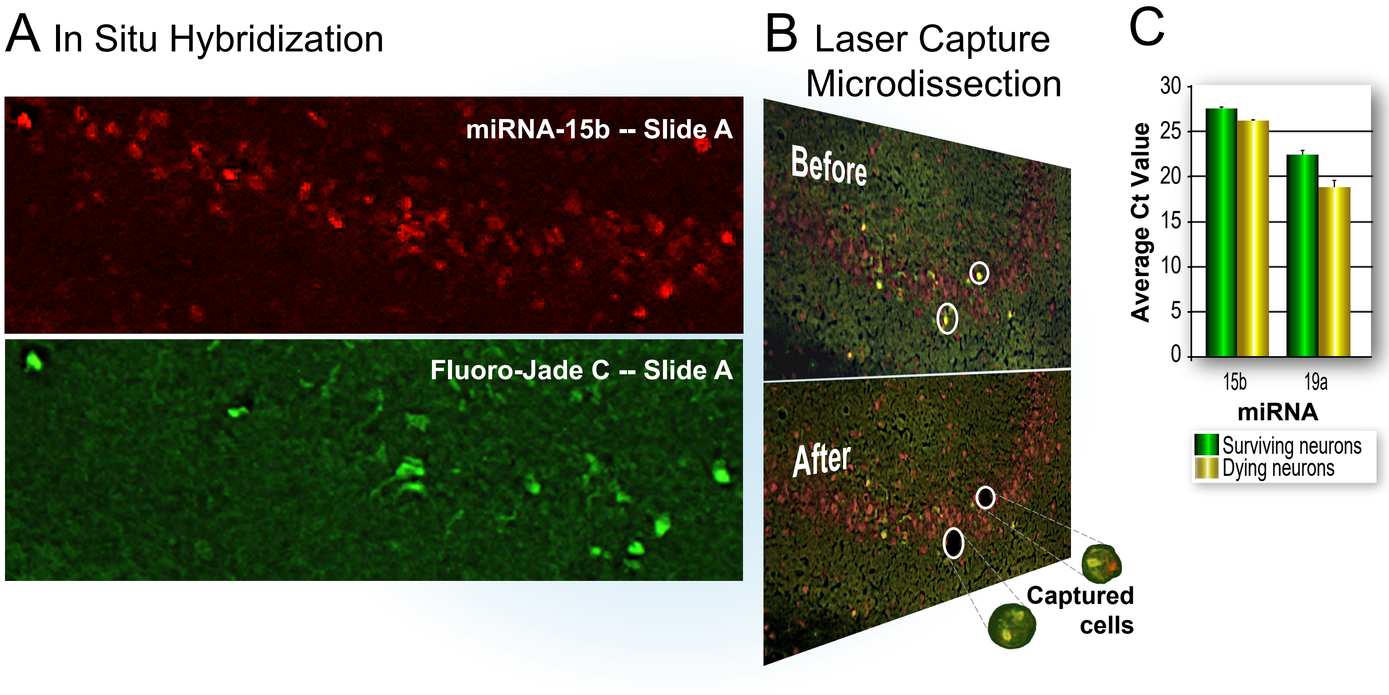

Supplementary Figure 6. Analysis of microRNA expression in pools (n=6) of 30 individual cells (dying, FJ+ or surviving FJ- neurons) using Fluidigm's microfluidic 48.48 Dynamic Array integrated fluid circuit microRNA PCR chip. Total RNA containing microRNA was isolated from laser capture microdissected dying, Fluoro-Jade-positive and miR-15b positive or surviving, Fluoro-Jade-negative and miR-15b negative neurons using the RNAqueous Micro Kit (Ambion). Reverse Transcription was performed using the RT primers provided in the TaqMan MegaPlex Primer Pool A and the microRNA Reverse Transcription Kit. The resulting cDNA was then pre-amplified using the pooled Preamp primers provided in the MegaPlex kit for 15-18 cycles. The preamped products were then diluted 1:10 and 40 cycles of PCR were performed on Biomark HD system (Fluidigm Corp. San Francisco, CA). The data was analyzed using Fluidigm Real-Time Data Analysis Software. Although not statistically significant, microfluidic PCR expression levels in dying or surviving neurons trend in the same direction (lower CT values correspond to higher expression) and correlate with in situ hybridization results (Fig. 3)

Supplementary Figure 7.

A

Rat BDNF ENST00000439476.2 3' UTR length: 2973

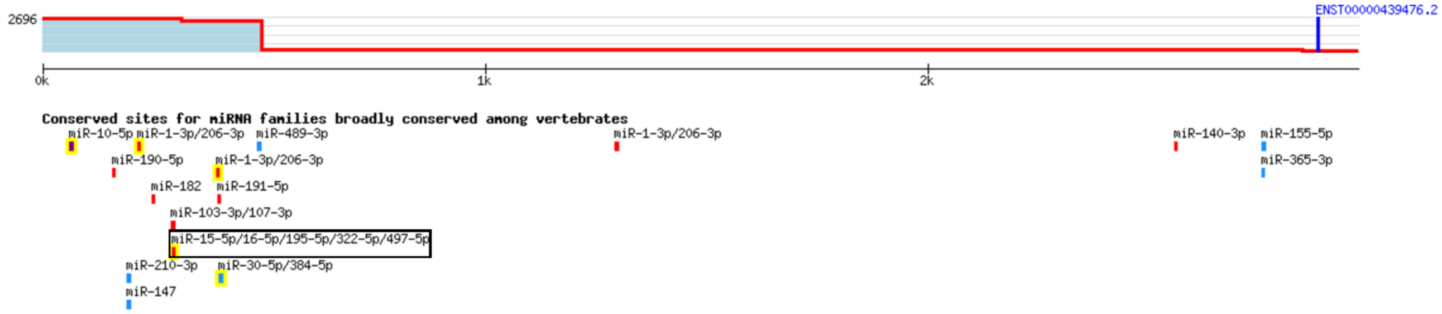

B

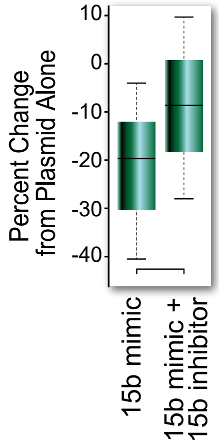

Supplementary Figure 7. Evidence that miR-15b regulates brain-derived neurotrophic factor (BDNF) mRNA expression. **A.** TargetScan analysis shows that the 3' untranslated region (UTR) of BDNF contains seed binding sites for multiple brain injury dysregulated miRNAs including miR-15b. **B.** Dual luciferase reporter assays using a GenoCopoeia *Bdnf* 3'UTR construct shows that a miR-15b mimic downregulates *Bdnf* expression and a miR-15b inhibitor sequesters the miR-15b mimic and returns *Bdnf*/luciferase expression closer to control levels. Bars represent 95% confidence intervals.

Supplementary References

References for microRNA gene targets in Supplementary Table 3

1. Conner,S.D. & Schmid,S.L. Identification of an adaptor-associated kinase, AAK1, as a regulator of clathrin-mediated endocytosis. J. Cell Biol. **156**, 921-929 (2002).

2. Toompuu,M., Karblane,K., Pata,P., Truve,E., & Sarmiento,C. ABCE1 is essential for S phase progression in human cells. *Cell Cycle* **15**, 1234-1247 (2016).

3. Graham,A. Mitochondrial regulation of macrophage cholesterol homeostasis. *Free Radic. Biol. Med.* **89**, 982-992 (2015).

4. Poppelreuther,M. *et al.* The N-terminal region of acyl-CoA synthetase 3 is essential for both the localization on lipid droplets and the function in fatty acid uptake. *J. Lipid Res.* **53**, 888-900 (2012).

5. Wu,X. *et al.* ACSL4 promotes prostate cancer growth, invasion and hormonal resistance. *Oncotarget.* **6**, 44849-44863 (2015).

6. Jiang,H.K., Qiu,G.R., Li-Ling,J., Xin,N., & Sun,K.L. Reduced ACTC1 expression might play a role in the onset of congenital heart disease by inducing cardiomyocyte apoptosis. *Circ. J.* **74**, 2410-2418 (2010).

7. Hodges,J.L., Vilchez,S.M., Asmussen,H., Whitmore,L.A., & Horwitz,A.R. alpha-Actinin-2 mediates spine morphology and assembly of the post-synaptic density in hippocampal neurons. *PLoS. One.* **9**, e101770 (2014).

8. Reglodi,D. *et al.* PACAP is an endogenous protective factor-insights from PACAP-deficient mice. *J Mol. Neurosci.* **48**, 482-492 (2012).

9. Wang,Y. *et al.* Ankrd17 positively regulates RIG-I-like receptor (RLR)-mediated immune signaling. *Eur. J. Immunol.* **42**, 1304-1315 (2012).

10. Huang,Y. *et al.* Down-regulated expression of Annexin A7 induces apoptosis in mouse hepatocarcinoma cell line by the intrinsic mitochondrial pathway. *Biomed. Pharmacother.* **70**, 146-150 (2015).

11. Laporte,S.A. *et al.* The beta2-adrenergic receptor/betaarrestin complex recruits the clathrin adaptor AP-2 during endocytosis. *Proc. Natl. Acad. Sci U. S. A* **96**, 3712-3717 (1999).

12. Ishizaki,H. *et al.* Combined zebrafish-yeast chemical-genetic screens reveal gene-copper-nutrition interactions that modulate melanocyte pigmentation. *Dis. Model. Mech.* **3**, 639-651 (2010).

13. Stanga,S. *et al.* APP-dependent glial cell line-derived neurotrophic factor gene expression drives neuromuscular junction formation. *FASEB J.* **30**, 1696-1711 (2015).

14. Li,S. *et al.* Remote ischemic post-conditioning improves neurological function by AQP4 down-regulation in astrocytes. *Behav. Brain Res.* **289**, 1-8 (2015).

15. Xu,X. *et al.* Mutation in archain 1, a subunit of COPI coatomer complex, causes diluted coat color and Purkinje cell degeneration. *PLoS. Genet.* **6**, e1000956 (2010).

16. Lovrecic,L., Slavkov,I., Dzeroski,S., & Peterlin,B. ADP-ribosylation factor guanine nucleotide-exchange factor 2 (ARFGEF2): a new potential biomarker in Huntington's disease. *J. Int. Med. Res.* **38**, 1653-1662 (2010).

17. Gonzalez-Munoz,E., Arboleda-Estudillo,Y., Otu,H.H., & Cibelli,J.B. Cell reprogramming. Histone chaperone ASF1A is required for maintenance of pluripotency and cellular reprogramming. *Science* **345**, 822-825 (2014).

18. Wilson,P.M., Fryer,R.H., Fang,Y., & Hatten,M.E. Astn2, a novel member of the astrotactin gene family, regulates the trafficking of ASTN1 during glial-guided neuronal migration. *J. Neurosci.* **30**, 8529-8540 (2010).

19. Wu,X. *et al.* Autophagy regulates Notch degradation and modulates stem cell development and neurogenesis. *Nat. Commun.* **7**, 10533 (2016).

20. Guo,D., Du,Y., Wu,Q., Jiang,W., & Bi,H. Disrupted calcium homeostasis is involved in elevated zinc ion-induced photoreceptor cell death. *Arch. Biochem. Biophys.* **560**, 44-51 (2014).

21. Eden,A. & Benvenisty,N. Involvement of branched-chain amino acid aminotransferase (Bcat1/Eca39) in apoptosis. *FEBS Lett.* **457**, 255-261 (1999).

22. Vollmayr,B., Faust,H., Lewicka,S., & Henn,F.A. Brain-derived-neurotrophic-factor (BDNF) stress response in rats bred for learned helplessness. *Mol. Psychiatry* **6**, 471-4, 358 (2001).

23. Mitra,P., Vaughan,P.S., Stein,J.L., Stein,G.S., & van Wijnen,A.J. Purification and functional analysis of a novel leucine-zipper/nucleotide-fold protein, BZAP45, stimulating cell cycle regulated histone H4 gene transcription. *Biochemistry* **40**, 10693-10699 (2001).

24. Muthana,M. *et al.* C5orf30 is a negative regulator of tissue damage in rheumatoid arthritis. *Proc. Natl. Acad. Sci U. S. A* **112**, 11618-11623 (2015).

25. Lee,A.S. *et al.* The Neuropsychiatric Disease-Associated Gene cacna1c Mediates Survival of Young Hippocampal Neurons. *eNeuro.* **3**, (2016).

26. Shiina,N., Yamaguchi,K., & Tokunaga,M. RNG105 deficiency impairs the dendritic localization of mRNAs for Na+/K+ ATPase subunit isoforms and leads to the degeneration of neuronal networks. *J. Neurosci.* **30**, 12816-12830 (2010).

27. Upton,J.P. *et al.* IRE1alpha cleaves select microRNAs during ER stress to derepress translation of proapoptotic Caspase-2. *Science* **338**, 818-822 (2012).

28. Yang,Q. *et al.* Sevoflurane preconditioning induces neuroprotection through reactive oxygen species-mediated up-regulation of antioxidant enzymes in rats. *Anesth. Analg.* **112**, 931-937 (2011).

29. Schlager,M.A. *et al.* Pericentrosomal targeting of Rab6 secretory vesicles by Bicaudal-D-related protein 1 (BICDR-1) regulates neuritogenesis. *EMBO J.* **29**, 1637-1651 (2010).

30. Liu,X. *et al.* MiR-1271 Inhibits Ovarian Cancer Growth by Targeting Cyclin G1. *Med. Sci. Monit.* **21**, 3152-3158 (2015).

31. Sun,T., Co,N.N., & Wong,N. PFTK1 interacts with cyclin Y to activate non-canonical Wnt signaling in hepatocellular carcinoma. *Biochem. Biophys. Res. Commun.* **449**, 163-168 (2014).

32. Chiang,I.T. *et al.* Curcumin alters gene expression-associated DNA damage, cell cycle, cell survival and cell migration and invasion in NCI-H460 human lung cancer cells in vitro. *Oncol. Rep.* **34**, 1853-1874 (2015).

33. Wu,F., Peacock,S.O., Rao,S., Lemmon,S.K., & Burnstein,K.L. Novel interaction between the co-chaperone Cdc37 and Rho GTPase exchange factor Vav3 promotes androgen receptor activity and prostate cancer growth. *J Biol. Chem* **288**, 5463-5474 (2013).

34. Gil,O.D., Needleman,L., & Huntley,G.W. Developmental patterns of cadherin expression and localization in relation to compartmentalized thalamocortical terminations in rat barrel cortex. *J. Comp Neurol.* **453**, 372-388 (2002).

35. Mekontso,D.A. *et al.* Ventilator-associated pneumonia during weaning from mechanical ventilation: role of fluid management. *Chest* **146**, 58-65 (2014).

36. Yang,X. *et al.* Gene reactivation by 5-aza-2'-deoxycytidine-induced demethylation requires SRCAP-mediated H2A.Z insertion to establish nucleosome depleted regions. *PLoS. Genet.* **8**, e1002604 (2012).

37. Singh,U. & Westermark,B. CGGBP1--an indispensable protein with ubiquitous cytoprotective functions. *Ups. J. Med. Sci* **120**, 219-232 (2015).

38. de,D.M. *et al.* Genome-wide nucleosome specificity and function of chromatin remodellers in ES cells. *Nature* **530**, 113-116 (2016).

39. McCullough,J. *et al.* Structure and membrane remodeling activity of ESCRT-III helical polymers. *Science* **350**, 1548-1551 (2015).

40. Fu,J. *et al.* TPX2 phosphorylation maintains metaphase spindle length by regulating microtubule flux. *J. Cell Biol.* **210**, 373-383 (2015).

41. Hu,H. *et al.* X-exome sequencing of 405 unresolved families identifies seven novel intellectual disability genes. *Mol. Psychiatry* **21**, 133-148 (2016).

42. Boudkkazi,S., Brechet,A., Schwenk,J., & Fakler,B. Cornichon2 dictates the time course of excitatory transmission at individual hippocampal synapses. *Neuron* **82**, 848-858 (2014).

43. de Baaij,J.H., Hoenderop,J.G., & Bindels,R.J. Magnesium in man: implications for health and disease. *Physiol Rev.* **95**, 1-46 (2015).

44. Sanchez-Blazquez,P., Rodriguez-Munoz,M., Vicente-Sanchez,A., & Garzon,J. Cannabinoid receptors couple to NMDA receptors to reduce the production of NO and the mobilization of zinc induced by glutamate. *Antioxid. Redox. Signal.* **19**, 1766-1782 (2013).

45. Su,H. *et al.* COP9 Signalosome Controls the Degradation of Cytosolic Misfolded Proteins and Protects Against Cardiac Proteotoxicity. *Circ. Res.* **117**, 956-966 (2015).

46. Barbaro,M., Kotajarvi,M., Harper,P., & Floderus,Y. Identification of an AluY-mediated deletion of exon 5 in the CPOX gene by MLPA analysis in patients with hereditary coproporphyria. *Clin. Genet.* **81**, 249-256 (2012).

47. Zhang,R., Thamm,D.H., & Misra,V. The effect of Zhangfei/CREBZF on cell growth, differentiation, apoptosis, migration, and the unfolded protein response in several canine osteosarcoma cell lines. *BMC. Vet. Res.* **11**, 22 (2015).

48. Kobayashi,H., Kawauchi,D., Hashimoto,Y., Ogata,T., & Murakami,F. The control of precerebellar neuron migration by RNA-binding protein Csde1. *Neuroscience* **253**, 292-303 (2013).

49. Zhu,J.H. *et al.* Suppression of cellular apoptosis susceptibility (CSE1L) inhibits proliferation and induces apoptosis in colorectal cancer cells. *Asian Pac. J. Cancer Prev.* **14**, 1017-1021 (2013).

50. Sun,T., Turk,V., Turk,B., & Kopitar-Jerala,N. Increased expression of stefin B in the nucleus of T98G astrocytoma cells delays caspase activation. *Front Mol. Neurosci.* **5**, 93 (2012).

51. Jimenez-Gonzalez,M. *et al.* Cardiotrophin 1 protects beta cells from apoptosis and prevents streptozotocin-induced diabetes in a mouse model. *Diabetologia* **56**, 838-846 (2013).

52. Fan,K. *et al.* Up-regulation of microglial cathepsin C expression and activity in lipopolysaccharide-induced neuroinflammation. *J. Neuroinflammation.* **9**, 96 (2012).

53. Chen,Y.K. & Hsueh,Y.P. Cortactin-binding protein 2 modulates the mobility of cortactin and regulates dendritic spine formation and maintenance. *J. Neurosci.* **32**, 1043-1055 (2012).

54. Zhao,Y. & Sun,Y. Cullin-RING Ligases as attractive anti-cancer targets. *Curr. Pharm. Des* **19**, 3215-3225 (2013).

55. Ritterhoff,S. *et al.* The WD40-repeat protein Han11 functions as a scaffold protein to control HIPK2 and MEKK1 kinase functions. *EMBO J.* **29**, 3750-3761 (2010).

56. Liguori,R. *et al.* Impairment of brain and muscle energy metabolism detected by magnetic resonance spectroscopy in hereditary spastic paraparesis type 28 patients with DDHD1 mutations. *J Neurol.* **261**, 1789-1793 (2014).

57. Chen,H.H., Yu,H.I., & Tarn,W.Y. DDX3 Modulates Neurite Development via Translationally Activating an RNA Regulon Involved in Rac1 Activation. *J. Neurosci.* **36**, 9792-9804 (2016).

58. Lee,H.J. *et al.* Genotoxic stress/p53-induced DNAJB9 inhibits the pro-apoptotic function of p53. *Cell Death. Differ.* **22**, 86-95 (2015).

59. Qiu,X.B., Shao,Y.M., Miao,S., & Wang,L. The diversity of the DnaJ/Hsp40 family, the crucial partners for Hsp70 chaperones. *Cell Mol. Life Sci.* **63**, 2560-2570 (2006).

60. Arque,G., Casanovas,A., & Dierssen,M. Dyrk1A is dynamically expressed on subsets of motor neurons and in the neuromuscular junction: possible role in Down syndrome. *PLoS. One.* **8**, e54285 (2013).

61. Chun,C. *et al.* The homologous recombination component EEPD1 is required for genome stability in response to developmental stress of vertebrate embryogenesis. *Cell Cycle* **15**, 957-962 (2016).

62. Stacey,D. *et al.* A translational systems biology approach in both animals and humans identifies a functionally related module of accumbal genes involved in the regulation of reward processing and binge drinking in males. *J. Psychiatry Neurosci.* **41**, 192-202 (2016).

63. Placzek,A.N. *et al.* Translational control of nicotine-evoked synaptic potentiation in mice and neuronal responses in human smokers by eIF2alpha. *Elife.* **5**, (2016).

64. Ozpolat,B. *et al.* Death-associated protein 5 (DAP5/p97/NAT1) contributes to retinoic acid-induced granulocytic differentiation and arsenic trioxide-induced apoptosis in acute promyelocytic leukemia. *Apoptosis.* **13**, 915-928 (2008).

65. Ando,M. *et al.* Mutational Landscape and Antiproliferative Functions of ELF Transcription Factors in Human Cancer. *Cancer Res.* **76**, 1814-1824 (2016).

66. Johnson,K.R., Longo-Guess,C.M., & Gagnon,L.H. Mutations of the mouse ELMO domain containing 1 gene (Elmod1) link small GTPase signaling to actin cytoskeleton dynamics in hair cell stereocilia. *PLoS. One.* **7**, e36074 (2012).

67. Peng,Y.R., Hou,Z.H., & Yu,X. The kinase activity of EphA4 mediates homeostatic scaling-down of synaptic strength via activation of Cdk5. *Neuropharmacology* **65**, 232-243 (2013).

68. Torii,M., Rakic,P., & Levitt,P. Role of EphA/ephrin--a signaling in the development of topographic maps in mouse corticothalamic projections. *J. Comp Neurol.* **521**, 626-637 (2013).

69. Bogliolo,M. *et al.* Mutations in ERCC4, encoding the DNA-repair endonuclease XPF, cause Fanconi anemia. *Am. J. Hum. Genet.* **92**, 800-806 (2013).

70. Linford,A. *et al.* Rab14 and its exchange factor FAM116 link endocytic recycling and adherens junction stability in migrating cells. *Dev. Cell* **22**, 952-966 (2012).

71. Honsho,M., Asaoku,S., Fukumoto,K., & Fujiki,Y. Topogenesis and homeostasis of fatty acyl-CoA reductase 1. *J. Biol. Chem.* **288**, 34588-34598 (2013).

72. Katoh,M. Function and cancer genomics of FAT family genes (review). *Int. J Oncol.* **41**, 1913-1918 (2012).

73. Gant,J.C. *et al.* Disrupting function of FK506-binding protein 1b/12.6 induces the Ca(2)+-dysregulation aging phenotype in hippocampal neurons. *J Neurosci.* **31**, 1693-1703 (2011).

74. Baudot,A.D. *et al.* p53 directly regulates the glycosidase FUCA1 to promote chemotherapy-induced cell death. *Cell Cycle* **15**, 2299-2308 (2016).

75. Wu,W. *et al.* FUNDC1 is a novel mitochondrial-associated-membrane (MAM) protein required for hypoxia-induced mitochondrial fission and mitophagy. *Autophagy.* **12**, 1675-1676 (2016).

76. Grabenstatter,H.L. *et al.* Effect of spontaneous seizures on GABAA receptor alpha4 subunit expression in an animal model of temporal lobe epilepsy. *Epilepsia* **55**, 1826-1833 (2014).

77. Weber,H. *et al.* Gender differences in associations of glutamate decarboxylase 1 gene (GAD1) variants with panic disorder. *PLoS. One.* **7**, e37651 (2012).

78. Chowdhury,A.H. *et al.* Differential transcriptional regulation of meis1 by Gfi1b and its co-factors LSD1 and CoREST. *PLoS. One.* **8**, e53666 (2013).

79. Florea,V. *et al.* Agonists of growth hormone-releasing hormone stimulate self-renewal of cardiac stem cells and promote their survival. *Proc. Natl. Acad. Sci U. S. A* **111**, 17260-17265 (2014).

80. de Vasconcellos,J.F. *et al.* SB225002 Induces Cell Death and Cell Cycle Arrest in Acute Lymphoblastic Leukemia Cells through the Activation of GLIPR1. *PLoS One* **10**, e0134783 (2015).

81. Qie,S., Chu,C., Li,W., Wang,C., & Sang,N. ErbB2 activation upregulates glutaminase 1 expression which promotes breast cancer cell proliferation. *J. Cell Biochem.* **115**, 498-509 (2014).

82. Li,D.D. *et al.* Inhibition of the oxidative stress-induced miR-23a protects the human retinal pigment epithelium (RPE) cells from apoptosis through the upregulation of glutaminase and glutamine uptake. *Mol. Biol. Rep.* **43**, 1079-1087 (2016).

83. Masuhara,M., Nagao,K., Nishikawa,M., Kimura,T., & Nakano,T. Enhanced degradation of MDM2 by a nuclear envelope component, mouse germ cell-less. *Biochem. Biophys. Res. Commun.* **308**, 927-932 (2003).

84. Carrigan,M. *et al.* A novel homozygous truncating GNAT1 mutation implicated in retinal degeneration. *Br. J. Ophthalmol.* **100**, 495-500 (2015).

85. Bakken,T.E. *et al.* Association of common genetic variants in GPCPD1 with scaling of visual cortical surface area in humans. *Proc. Natl. Acad. Sci. U. S. A* **109**, 3985-3990 (2012).

86. Trousil,S. *et al.* Alterations of choline phospholipid metabolism in endometrial cancer are caused by choline kinase alpha overexpression and a hyperactivated deacylation pathway. *Cancer Res.* **74**, 6867-6877 (2014).

87. Michibata,H. *et al.* Human GPM6A is associated with differentiation and neuronal migration of neurons derived from human embryonic stem cells. *Stem Cells Dev.* **18**, 629-639 (2009).

88. Tuszynski,G.P. *et al.* G-protein coupled receptor-associated sorting protein 1 (GASP-1), a potential biomarker in breast cancer. *Exp. Mol. Pathol.* **91**, 608-613 (2011).

89. Umemori,J. *et al.* ENU-mutagenesis mice with a non-synonymous mutation in Grin1 exhibit abnormal anxiety-like behaviors, impaired fear memory, and decreased acoustic startle response. *BMC. Res. Notes* **6**, 203 (2013).

90. Ma,Y. *et al.* NAD+ Metabolism and NAD+-Dependent Enzymes: Promising Therapeutic Targets for Neurological Diseases. *Curr. Drug Targets.* **13**, 222-229 (2012).

91. Kainulainen,M. *et al.* Virulence factor NSs of rift valley fever virus recruits the F-box protein FBXO3 to degrade subunit p62 of general transcription factor TFIIH. *J. Virol.* **88**, 3464-3473 (2014).

92. Alfaiz,A.A. *et al.* West syndrome caused by homozygous variant in the evolutionary conserved gene encoding the mitochondrial elongation factor GUF1. *Eur. J. Hum. Genet.* **24**, 1001-1008 (2016).

93. Vester,A. *et al.* A loss-of-function variant in the human histidyl-tRNA synthetase (HARS) gene is neurotoxic in vivo. *Hum. Mutat.* **34**, 191-199 (2013).

94. Du,L. *et al.* Rumba and Haus3 are essential factors for the maintenance of hematopoietic stem/progenitor cells during zebrafish hematopoiesis. *Development* **138**, 619-629 (2011).

95. Dowejko,A., Bauer,R., Bauer,K., Muller-Richter,U.D., & Reichert,T.E. The human HECA interacts with cyclins and CDKs to antagonize Wnt-mediated proliferation and chemoresistance of head and neck cancer cells. *Exp. Cell Res.* **318**, 489-499 (2012).

96. Sakamoto,M., Hirata,H., Ohtsuka,T., Bessho,Y., & Kageyama,R. The basic helix-loop-helix genes Hesr1/Hey1 and Hesr2/Hey2 regulate maintenance of neural precursor cells in the brain. *J Biol. Chem* **278**, 44808-44815 (2003).

97. Gravel,R.A. Holocarboxylase synthetase: a multitalented protein with roles in biotin transfer, gene regulation and chromatin dynamics. *Mol. Genet. Metab* **111**, 305-306 (2014).

98. Pandyra,A.A. *et al.* Genome-wide RNAi analysis reveals that simultaneous inhibition of specific mevalonate pathway genes potentiates tumor cell death. *Oncotarget.* **6**, 26909-26921 (2015).

99. Mori,K. *et al.* Reduced hnRNPA3 increases C9orf72 repeat RNA levels and dipeptide-repeat protein deposition. *EMBO Rep.* **17**, 1314-1325 (2016).

100. Sadri,N. & Schneider,R.J. Auf1/Hnrnpd-deficient mice develop pruritic inflammatory skin disease. *J. Invest Dermatol.* **129**, 657-670 (2009).

101. Ye,J. *et al.* hnRNP U protein is required for normal pre-mRNA splicing and postnatal heart development and function. *Proc. Natl. Acad. Sci. U. S. A* **112**, E3020-E3029 (2015).

102. Mace,K.A., Hansen,S.L., Myers,C., Young,D.M., & Boudreau,N. HOXA3 induces cell migration in endothelial and epithelial cells promoting angiogenesis and wound repair. *J Cell Sci.* **118**, 2567-2577 (2005).

103. Garfinkel,B.P., Melamed-Book,N., Anuka,E., Bustin,M., & Orly,J. HP1BP3 is a novel histone H1 related protein with essential roles in viability and growth. *Nucleic Acids Res.* **43**, 2074-2090 (2015).

104. Nguyen,K.V. & Nyhan,W.L. Mutation in the Human HPRT1 Gene and the Lesch-Nyhan Syndrome. *Nucleosides Nucleotides Nucleic Acids* **35**, 426-433 (2016).

105. Nakano,T. *et al.* Establishment of a human lung cancer cell line with high metastatic potential to multiple organs: gene expression associated with metastatic potential in human lung cancer. *Oncol. Rep.* **28**, 1727-1735 (2012).

106. Schneider,R., Linka,R.M., & Reinke,H. HSP90 affects the stability of BMAL1 and circadian gene expression. *J. Biol. Rhythms* **29**, 87-96 (2014).

107. Shirakihara,T. *et al.* Identification of integrin alpha3 as a molecular marker of cells undergoing epithelial-mesenchymal transition and of cancer cells with aggressive phenotypes. *Cancer Sci.* **104**, 1189-1197 (2013).

108. Park,B.S., Yeo,S.G., Jung,J., & Jeong,N.Y. A novel therapeutic target for peripheral nerve injury-related diseases: aminoacyl-tRNA synthetases. *Neural Regen. Res.* **10**, 1656-1662 (2015).

109. Cavallaro,S. Cracking the code of neuronal apoptosis and survival. *Cell Death. Dis.* **6**, e1963 (2015).

110. Tcherpakov,M. *et al.* JAMP optimizes ERAD to protect cells from unfolded proteins. *Mol. Biol. Cell* **19**, 5019-5028 (2008).

111. Sun,G. & Irvine,K.D. Ajuba family proteins link JNK to Hippo signaling. *Sci. Signal.* **6**, ra81 (2013).

112. Zhang,Y. *et al.* Gene Mutation Analysis in 253 Chinese Children with Unexplained Epilepsy and Intellectual/Developmental Disabilities. *PLoS. One.* **10**, e0141782 (2015).

113. Cadet,J.L. *et al.* Genome-wide DNA hydroxymethylation identifies potassium channels in the nucleus accumbens as discriminators of methamphetamine addiction and abstinence. *Mol. Psychiatry*(2016). [Epub ahead of print]

114. Poirier,K. *et al.* Mutations in TUBG1, DYNC1H1, KIF5C and KIF2A cause malformations of cortical development and microcephaly. *Nat. Genet.* **45**, 639-647 (2013).

115. Yu,X. *et al.* Temporal and spatial expression of KIF3B after acute spinal cord injury in adult rats. *J Mol. Neurosci.* **49**, 387-394 (2013).

116. Tanaka,M. *et al.* EVI1 oncogene promotes KRAS pathway through suppression of microRNA-96 in pancreatic carcinogenesis. *Oncogene* **33**, 2454-2463 (2014).

117. Cronan,J.E. Biotin and Lipoic Acid: Synthesis, Attachment, and Regulation. *EcoSal. Plus.* **6**, (2014).

118. You,X. *et al.* Hepatitis B virus X protein upregulates Lin28A/Lin28B through Sp-1/c-Myc to enhance the proliferation of hepatoma cells. *Oncogene* **33**, 449-460 (2014).

119. Yu,P. *et al.* Cooperative interactions of LPPR family members in membrane localization and alteration of cellular morphology. *J. Cell Sci* **128**, 3210-3222 (2015).

120. Strauss,U. & Brauer,A.U. Current views on regulation and function of plasticity-related genes (PRGs/LPPRs) in the brain. *Biochim. Biophys. Acta* **1831**, 133-138 (2013).

121. Kochunov,P. *et al.* Transcriptomics of cortical gray matter thickness decline during normal aging. *Neuroimage.* **82**, 273-283 (2013).

122. Minatohara,K., Murata,Y., Fujiyoshi,Y., & Doi,T. An intracellular domain with a novel sequence regulates cell surface expression and synaptic clustering of leucine-rich repeat transmembrane proteins in hippocampal neurons. *J. Neurochem.* **134**, 618-628 (2015).

123. Reed,M., Morris,S.H., Owczarczyk,A.B., & Lukacs,N.W. Deficiency of autophagy protein Map1-LC3b mediates IL-17-dependent lung pathology during respiratory viral infection via ER stress-associated IL-1. *Mucosal. Immunol.* **8**, 1118-1130 (2015).

124. Wu,H. *et al.* Combination of early and delayed ischemic postconditioning enhances brain-derived neurotrophic factor production by upregulating the ERK-CREB pathway in rats with focal ischemia. *Mol. Med. Rep.* **12**, 6427-6434 (2015).

125. Brown,M. *et al.* An initial phase of JNK activation inhibits cell death early in the endoplasmic reticulum stress response. *J. Cell Sci* **129**, 2317-2328 (2016).

126. Watanabe,T. *et al.* TTBK2 with EB1/3 regulates microtubule dynamics in migrating cells through KIF2A phosphorylation. *J. Cell Biol.* **210**, 737-751 (2015).

127. Trovo,L., Stroobants,S., D'Hooge,R., Ledesma,M.D., & Dotti,C.G. Improvement of biochemical and behavioral defects in the Niemann-Pick type A mouse by intraventricular infusion of MARCKS. *Neurobiol. Dis.* **73**, 319-326 (2015).

128. Wu,P.R. *et al.* DAPK activates MARK1/2 to regulate microtubule assembly, neuronal differentiation, and tau toxicity. *Cell Death. Differ.* **18**, 1507-1520 (2011).

129. Jonas,A. *et al.* Axonally derived matrilin-2 induces proinflammatory responses that exacerbate autoimmune neuroinflammation. *J Clin. Invest* **124**, 5042-5056 (2014).

130. Jung,J.S. *et al.* MBD6 is a direct target of Oct4 and controls the stemness and differentiation of adipose tissue-derived stem cells. *Cell Mol. Life Sci* **70**, 711-728 (2013).

131. Keembiyehetty,C. *et al.* Conditional knock-out reveals a requirement for O-linked N-Acetylglucosaminase (O-GlcNAcase) in metabolic homeostasis. *J. Biol. Chem.* **290**, 7097-7113 (2015).

132. Bernal,F., Hartung,H.P., & Kieseier,B.C. Tissue mRNA expression in rat of newly described matrix metalloproteinases. *Biol. Res.* **38**, 267-271 (2005).

133. Xu,Q. *et al.* Expression profile of mouse Mterfd2, a novel component of the mitochondrial transcription termination factor (MTERF) family. *Genes Genet. Syst.* **86**, 269-275 (2011).

134. Taoka,M. *et al.* V-1, a protein expressed transiently during murine cerebellar development, regulates actin polymerization via interaction with capping protein. *J. Biol. Chem.* **278**, 5864-5870 (2003).

135. Murphy,D.M. *et al.* Dissection of the oncogenic MYCN transcriptional network reveals a large set of clinically relevant cell cycle genes as drivers of neuroblastoma tumorigenesis. *Mol. Carcinog.* **50**, 403-411 (2011).

136. Sui,W.H. *et al.* Myosin Va mediates BDNF-induced postendocytic recycling of full-length TrkB and its translocation into dendritic spines. *J. Cell Sci.* **128**, 1108-1122 (2015).

137. Kravtsov,D. *et al.* Myosin 5b loss of function leads to defects in polarized signaling: implication for microvillus inclusion disease pathogenesis and treatment. *Am. J Physiol Gastrointest. Liver Physiol* **307**, G992-G1001 (2014).

138. Wang,X., Li,H., & Ding,S. Pre-B-cell colony-enhancing factor protects against apoptotic neuronal death and mitochondrial damage in ischemia. *Sci Rep.* **6**, 32416 (2016).

139. Inoue,H. *et al.* gamma-SNAP stimulates disassembly of endosomal SNARE complexes and regulates endocytic trafficking pathways. *J. Cell Sci* **128**, 2781-2794 (2015).

140. Attia,M., Rachez,C., De,P.A., Avner,P., & Rogner,U.C. Nap1l2 promotes histone acetylation activity during neuronal differentiation. *Mol. Cell Biol.* **27**, 6093-6102 (2007).

141. McNeill,E.M. *et al.* Nav2 hypomorphic mutant mice are ataxic and exhibit abnormalities in cerebellar development. *Dev. Biol.* **353**, 331-343 (2011).

142. Liu,M., Huangfu,X., Zhao,Y., Zhang,D., & Zhang,J. Steroid receptor coactivator-1 mediates letrozole induced downregulation of postsynaptic protein PSD-95 in the hippocampus of adult female rats. *J. Steroid Biochem. Mol. Biol.* **154**, 168-175 (2015).

143. Wu,Z. *et al.* Role of nuclear receptor coactivator 3 (Ncoa3) in pluripotency maintenance. *J. Biol. Chem.* **287**, 38295-38304 (2012).

144. Innocenti,M. *et al.* Abi1 is essential for the formation and activation of a WAVE2 signalling complex. *Nat Cell Biol.* **6**, 319-327 (2004).

145. Chen,Y. *et al.* Norrin protected blood-brain barrier via frizzled-4/beta-catenin pathway after subarachnoid hemorrhage in rats. *Stroke* **46**, 529-536 (2015).

146. Tang,M., Ivakine,E., Mahadevan,V., Salter,M.W., & McInnes,R.R. Neto2 interacts with the scaffolding protein GRIP and regulates synaptic abundance of kainate receptors. *PLoS One* **7**, e51433 (2012).

147. Ebel,J., Beuter,S., Wuchter,J., Kriebel,M., & Volkmer,H. Organisation and control of neuronal connectivity and myelination by cell adhesion molecule neurofascin. *Adv. Neurobiol.* **8**, 231-247 (2014).

148. Berga-Bolanos,R., Alberdi,M., Buxade,M., Aramburu,J., & Lopez-Rodriguez,C. NFAT5 induction by the pre-T-cell receptor serves as a selective survival signal in T-lymphocyte development. *Proc. Natl. Acad. Sci. U. S. A* **110**, 16091-16096 (2013).

149. Benatti,P. *et al.* Specific inhibition of NF-Y subunits triggers different cell proliferation defects. *Nucleic Acids Res.* **39**, 5356-5368 (2011).

150. El,W.B., Buhler,E., Haddad,M.R., & Villard,L. Nhej1 Deficiency Causes Abnormal Development of the Cerebral Cortex. *Mol. Neurobiol.* **52**, 771-782 (2015).

151. Tristan-Clavijo,E. *et al.* A truncating mutation in Alzheimer's disease inactivates neuroligin-1 synaptic function. *Neurobiol. Aging* **36**, 3171-3175 (2015).

152. Ishitani,T. & Ishitani,S. Nemo-like kinase, a multifaceted cell signaling regulator. *Cell Signal.* **25**, 190-197 (2013).

153. Bai,B., Moore,H.M., & Laiho,M. CRM1 and its ribosome export adaptor NMD3 localize to the nucleolus and affect rRNA synthesis. *Nucleus.* **4**, 315-325 (2013).

154. De,F.F. *et al.* Notch signaling sustains the expression of Mcl-1 and the activity of eIF4E to promote cell survival in CLL. *Oncotarget.* **6**, 16559-16572 (2015).

155. Musiek,E.S. *et al.* Circadian clock proteins regulate neuronal redox homeostasis and neurodegeneration. *J Clin. Invest* **123**, 5389-5400 (2013).

156. Bhattacharya,S. *et al.* Genetically Induced Retrograde Amnesia of Associative Memories After Neuroplastin Ablation. *Biol. Psychiatry*(2016). [Epub ahead of print]

157. Boles,N.C. *et al.* NPTX1 regulates neural lineage specification from human pluripotent stem cells. *Cell Rep.* **6**, 724-736 (2014).

158. McGowan,P.O. *et al.* Epigenetic regulation of the glucocorticoid receptor in human brain associates with childhood abuse. *Nat. Neurosci.* **12**, 342-348 (2009).

159. Reynolds,M.S. *et al.* beta-Cell deletion of Nr4a1 and Nr4a3 nuclear receptors impedes mitochondrial respiration and insulin secretion. *Am. J. Physiol Endocrinol. Metab* **311**, E186-E201 (2016).

160. Zito,A. *et al.* Neuritin 1 promotes neuronal migration. *Brain Struct. Funct.* **219**, 105-118 (2014).

161. Viphakone,N. *et al.* TREX exposes the RNA-binding domain of Nxf1 to enable mRNA export. *Nat. Commun.* **3**, 1006 (2012).

162. Wu,G. *et al.* Suppression of intestinal calcium entry channel TRPV6 by OCRL, a lipid phosphatase associated with Lowe syndrome and Dent disease. *Am. J Physiol Cell Physiol* **302**, C1479-C1491 (2012).

163. He,S., Liu,M., Zhang,W., Xu,N., & Zhu,H. Over expression of p21-activated kinase 7 associates with lymph node metastasis in esophageal squamous cell cancers. *Cancer Biomarkers* **16**, 203-209 (2016).

164. Kinjo,E.R. *et al.* Pilocarpine-induced seizures trigger differential regulation of microRNA-stability related genes in rat hippocampal neurons. *Sci Rep.* **6**, 20969 (2016).

165. Alarcon,V.B. Cell polarity regulator PARD6B is essential for trophectoderm formation in the preimplantation mouse embryo. *Biol. Reprod.* **83**, 347-358 (2010).

166. Macher-Goeppinger,S. *et al.* The PBRM1 (BAF180) protein is functionally regulated by p53-induced protein degradation in renal cell carcinomas. *J Pathol.*(2015).

167. Noguchi,Y. *et al.* Total expression and dual gene-regulatory mechanisms maintained in deletions and duplications of the Pcdha cluster. *J. Biol. Chem.* **284**, 32002-32014 (2009).

168. Morbt,N. *et al.* Chlorinated benzenes cause concomitantly oxidative stress and induction of apoptotic markers in lung epithelial cells (A549) at nonacute toxic concentrations. *J Proteome. Res.* **10**, 363-378 (2011).

169. Krause,C., Rosewich,H., Woehler,A., & Gartner,J. Functional analysis of PEX13 mutation in a Zellweger syndrome spectrum patient reveals novel homooligomerization of PEX13 and its role in human peroxisome biogenesis. *Hum. Mol. Genet.* **22**, 3844-3857 (2013).

170. Agostini,M. *et al.* Metabolic reprogramming during neuronal differentiation. *Cell Death. Differ.* **23**, 1502-1514 (2016).

171. Veiga-da-Cunha,M., Vleugels,W., Maliekal,P., Matthijs,G., & Van,S.E. Mammalian phosphomannomutase PMM1 is the brain IMP-sensitive glucose-1,6-bisphosphatase. *J Biol. Chem* **283**, 33988-33993 (2008).

172. Petersen,S.L. *et al.* Novel progesterone receptors: neural localization and possible functions. *Front Neurosci.* **7**, 164 (2013).

173. Siriwardana,N.S., Meyer,R., Havasi,A., Dominguez,I., & Panchenko,M.V. Cell cycle-dependent chromatin shuttling of HBO1-JADE1 histone acetyl transferase (HAT) complex. *Cell Cycle* **13**, 1885-1901 (2014).

174. Pei,D. *et al.* Distinct neuroblastoma-associated alterations of PHOX2B impair sympathetic neuronal differentiation in zebrafish models. *PLoS. Genet.* **9**, e1003533 (2013).

175. Perrone,R.D., Malek,A.M., & Watnick,T. Vascular complications in autosomal dominant polycystic kidney disease. *Nat. Rev. Nephrol.* **11**, 589-598 (2015).

176. Wu,C. *et al.* Mechano-sensitive PPAP2B regulates endothelial responses to athero-relevant hemodynamic forces. *Circ. Res.* **117**, e41-e53 (2015).

177. Lu,G. *et al.* A novel mitochondrial matrix serine/threonine protein phosphatase regulates the mitochondria permeability transition pore and is essential for cellular survival and development. *Genes Dev.* **21**, 784-796 (2007).

178. Fujimura,L. & Hatano,M. Role of Prickle1 and Prickle2 in neurite outgrowth in murine neuroblastoma cells. *Methods Mol. Biol.* **839**, 173-185 (2012).

179. Asad,M. *et al.* FZD7 drives in vitro aggressiveness in Stem-A subtype of ovarian cancer via regulation of non-canonical Wnt/PCP pathway. *Cell Death. Dis.* **5**, e1346 (2014).

180. de Moraes,D.C., Vaisman,M., Conceicao,F.L., & Ortiga-Carvalho,T.M. Pituitary development: a complex, temporal regulated process dependent on specific transcriptional factors. *J. Endocrinol.* **215**, 239-245 (2012).

181. Ghannam,K. *et al.* Upregulation of immunoproteasome subunits in myositis indicates active inflammation with involvement of antigen presenting cells, CD8 T-cells and IFNGamma. *PLoS. One.* **9**, e104048 (2014).

182. Wang,X., Bao,X., Pal,R., Agbas,A., & Michaelis,E.K. Transcriptomic responses in mouse brain exposed to chronic excess of the neurotransmitter glutamate. *BMC. Genomics* **11**, 360 (2010).

183. Zhi,H.Y. *et al.* PTPH1 cooperates with vitamin D receptor to stimulate breast cancer growth through their mutual stabilization. *Oncogene* **30**, 1706-1715 (2011).

184. Yuan,T., Wang,Y., Zhao,Z.J., & Gu,H. Protein-tyrosine phosphatase PTPN9 negatively regulates ErbB2 and epidermal growth factor receptor signaling in breast cancer cells. *J. Biol. Chem.* **285**, 14861-14870 (2010).

185. Nottingham,R.M. *et al.* RUTBC2 protein, a Rab9A effector and GTPase-activating protein for Rab36. *J. Biol. Chem.* **287**, 22740-22748 (2012).

186. Krasikova,Y.S. *et al.* Comparative analysis of interaction of human and yeast DNA damage recognition complexes with damaged DNA in nucleotide excision repair. *J. Biol. Chem.* **288**, 10936-10947 (2013).

187. Carmona,G. *et al.* Role of the small GTPase Rap1 for integrin activity regulation in endothelial cells and angiogenesis. *Blood* **113**, 488-497 (2009).

188. Schurmans,S., Polizzi,S., Scoumanne,A., Sayyed,S., & Molina-Ortiz,P. The Ras/Rap GTPase activating protein RASA3: from gene structure to in vivo functions. *Adv. Biol. Regul.* **57**, 153-161 (2015).

189. Lee,J.A. *et al.* Cytoplasmic Rbfox1 Regulates the Expression of Synaptic and Autism-Related Genes. *Neuron* **89**, 113-128 (2016).

190. Hashimoto,Y. *et al.* Protrudin regulates endoplasmic reticulum morphology and function associated with the pathogenesis of hereditary spastic paraplegia. *J Biol. Chem* **289**, 12946-12961 (2014).

191. Kwon,T. *et al.* Identifying direct targets of transcription factor Rfx2 that coordinate ciliogenesis and cell movement. *Genom. Data* **2**, 192-194 (2014).

192. Karelina,K. *et al.* Mitogen and stress-activated kinases 1/2 regulate ischemia-induced hippocampal progenitor cell proliferation and neurogenesis. *Neuroscience* **285**, 292-302 (2015).

193. Efeyan,A. *et al.* Regulation of mTORC1 by the Rag GTPases is necessary for neonatal autophagy and survival. *Nature* **493**, 679-683 (2013).

194. Shi,Q. *et al.* Impact of RTN3 deficiency on expression of BACE1 and amyloid deposition. *J. Neurosci.* **34**, 13954-13962 (2014).

195. Dickendesher,T.L. *et al.* NgR1 and NgR3 are receptors for chondroitin sulfate proteoglycans. *Nat. Neurosci.* **15**, 703-712 (2012).

196. Zheng,J.C. *et al.* Secretory Carrier Membrane Protein (SCAMP) deficiency influences behavior of adult flies. *Front Cell Dev. Biol.* **2**, 64 (2014).

197. Lossin,C. *et al.* Epilepsy-associated dysfunction in the voltage-gated neuronal sodium channel SCN1A. *J Neurosci.* **23**, 11289-11295 (2003).

198. Liao,Y. *et al.* SCN2A mutation associated with neonatal epilepsy, late-onset episodic ataxia, myoclonus, and pain. *Neurology* **75**, 1454-1458 (2010).

199. Itoh,Y. *et al.* Scratch regulates neuronal migration onset via an epithelial-mesenchymal transition-like mechanism. *Nat. Neurosci.* **16**, 416-425 (2013).

200. Ishii,N., Ishii,T., & Hartman,P.S. The role of the electron transport SDHC gene on lifespan and cancer. *Mitochondrion.* **7**, 24-28 (2007).

201. Li,H., Wu,D.K., & Sullivan,S.L. Characterization and expression of sema4g, a novel member of the semaphorin gene family. *Mech. Dev.* **87**, 169-173 (1999).

202. Miehe,S. *et al.* The phospholipid-binding protein SESTD1 is a novel regulator of the transient receptor potential channels TRPC4 and TRPC5. *J. Biol. Chem.* **285**, 12426-12434 (2010).

203. Gokmen-Polar,Y. *et al.* Expression levels of SF3B3 correlate with prognosis and endocrine resistance in estrogen receptor-positive breast cancer. *Mod. Pathol.* **28**, 677-685 (2015).

204. Meng,X. *et al.* Effects of overexpression of Sim2 on spatial memory and expression of synapsin I in rat hippocampus. *Cell Biol. Int.* **30**, 841-847 (2006).

205. Stromme,P. *et al.* X-linked Angelman-like syndrome caused by Slc9a6 knockout in mice exhibits evidence of endosomal-lysosomal dysfunction. *Brain* **134**, 3369-3383 (2011).

206. Orlowski,J. & Grinstein,S. Diversity of the mammalian sodium/proton exchanger SLC9 gene family. *Pflugers Arch.* **447**, 549-565 (2004).

207. Schuhmacher,A. *et al.* A variant of the neuronal amino acid transporter SLC6A15 is associated with ACTH and cortisol responses and cognitive performance in unipolar depression. *Int. J. Neuropsychopharmacol.* **16**, 83-90 (2013).

208. Langston,R.E. & Weinert,T. Nifty Alleles, a Plethora of Interactions, and Imagination Advance Understanding of Smc5/6's Roles with Chromosomes. *Mol. Cell* **60**, 832-833 (2015).

209. Zhang,Z. *et al.* The SNARE proteins SNAP25 and synaptobrevin are involved in endocytosis at hippocampal synapses. *J. Neurosci.* **33**, 9169-9175 (2013).

210. Turunen,J.J., Will,C.L., Grote,M., Luhrmann,R., & Frilander,M.J. The U11-48K protein contacts the 5' splice site of U12-type introns and the U11-59K protein. *Mol. Cell Biol.* **28**, 3548-3560 (2008).

211. Lin,Y.J. *et al.* Sorting nexin 24 genetic variation associates with coronary artery aneurysm severity in Kawasaki disease patients. *Cell Biosci.* **3**, 44 (2013).

212. Shibuya,Y., Chang,C.C., & Chang,T.Y. ACAT1/SOAT1 as a therapeutic target for Alzheimer's disease. *Future Med. Chem.* **7**, 2451-2467 (2015).

213. Kouri,N. *et al.* Genome-wide association study of corticobasal degeneration identifies risk variants shared with progressive supranuclear palsy. *Nat. Commun.* **6**, 7247 (2015).

214. Shu,Y.J. *et al.* SPOCK1 as a potential cancer prognostic marker promotes the proliferation and metastasis of gallbladder cancer cells by activating the PI3K/AKT pathway. *Mol. Cancer* **14**, 12 (2015).

215. Li,C.L., Sathyamurthy,A., Oldenborg,A., Tank,D., & Ramanan,N. SRF phosphorylation by glycogen synthase kinase-3 promotes axon growth in hippocampal neurons. *J Neurosci.* **34**, 4027-4042 (2014).

216. van Nues,R.W., Leung,E., McDonald,J.C., Dantuluru,I., & Brown,J.D. Roles for Srp72p in assembly, nuclear export and function of the signal recognition particle. *RNA. Biol.* **5**, 73-83 (2008).

217. Gruszczynska-Biegala,J. & Kuznicki,J. Native STIM2 and ORAI1 proteins form a calcium-sensitive and thapsigargin-insensitive complex in cortical neurons. *J. Neurochem.* **126**, 727-738 (2013).

218. Cinar,B. *et al.* The pro-apoptotic kinase Mst1 and its caspase cleavage products are direct inhibitors of Akt1. *EMBO J* **26**, 4523-4534 (2007).

219. Balatoni,C.E. *et al.* Epigenetic silencing of Stk39 in B-cell lymphoma inhibits apoptosis from genotoxic stress. *Am. J Pathol.* **175**, 1653-1661 (2009).

220. Price,M.P., Thompson,R.J., Eshcol,J.O., Wemmie,J.A., & Benson,C.J. Stomatin modulates gating of acid-sensing ion channels. *J. Biol. Chem.* **279**, 53886-53891 (2004).

221. Crevecoeur,J. *et al.* Expression pattern of synaptic vesicle protein 2 (SV2) isoforms in patients with temporal lobe epilepsy and hippocampal sclerosis. *Neuropathol. Appl. Neurobiol.* **40**, 191-204 (2014).

222. Bieniossek,C. *et al.* The architecture of human general transcription factor TFIID core complex. *Nature* **493**, 699-702 (2013).

223. Hulpke,S. *et al.* Direct evidence that the N-terminal extensions of the TAP complex act as autonomous interaction scaffolds for the assembly of the MHC I peptide-loading complex. *Cell Mol. Life Sci.* **69**, 3317-3327 (2012).

224. Han,B. *et al.* The role of TGFBI (betaig-H3) in gastrointestinal tract tumorigenesis. *Mol. Cancer* **14**, 64 (2015).

225. Ellenbroek,S.I., Iden,S., & Collard,J.G. The Rac activator Tiam1 is required for polarized protrusional outgrowth of primary astrocytes by affecting the organization of the microtubule network. *Small GTPases.* **3**, 4-14 (2012).

226. Smith-Roe,S.L. *et al.* Separation of intra-S checkpoint protein contributions to DNA replication fork protection and genomic stability in normal human fibroblasts. *Cell Cycle* **12**, 332-345 (2013).

227. Hong,G.S. *et al.* Tentonin 3/TMEM150c Confers Distinct Mechanosensitive Currents in Dorsal-Root Ganglion Neurons with Proprioceptive Function. *Neuron* **91**, 107-118 (2016).

228. Fujitomo,T., Daigo,Y., Matsuda,K., Ueda,K., & Nakamura,Y. Critical function for nuclear envelope protein TMEM209 in human pulmonary carcinogenesis. *Cancer Res.* **72**, 4110-4118 (2012).

229. Sunryd,J.C. *et al.* TMTC1 and TMTC2 are novel endoplasmic reticulum tetratricopeptide repeat-containing adapter proteins involved in calcium homeostasis. *J Biol. Chem* **289**, 16085-16099 (2014).

230. Sohn,W.J. *et al.* Expression and characterization of transmembrane and coiled-coil domain family 3. *BMB. Rep.*(2016). [Epub ahead of print]

231. Zhang,C.L. *et al.* Knockdown of TNFAIP1 inhibits growth and induces apoptosis in osteosarcoma cells through inhibition of the nuclear factor-kappaB pathway. *Oncol. Rep.* **32**, 1149-1155 (2014).

232. Kundu,J. *et al.* Tob1 induces apoptosis and inhibits proliferation, migration and invasion of gastric cancer cells by activating Smad4 and inhibiting betacatenin signaling. *Int. J. Oncol.* **41**, 839-848 (2012).

233. Wu,Y. *et al.* PIG11 is involved in hepatocellular carcinogenesis and its over-expression promotes Hepg2 cell apoptosis. *Pathol. Oncol. Res.* **15**, 411-416 (2009).

234. Thompson,S. *et al.* Identification of a novel Bcl-2-interacting mediator of cell death (Bim) E3 ligase, tripartite motif-containing protein 2 (TRIM2), and its role in rapid ischemic tolerance-induced neuroprotection. *J. Biol. Chem.* **286**, 19331-19339 (2011).

235. Zhou,Z. *et al.* TRIM14 is a mitochondrial adaptor that facilitates retinoic acid-inducible gene-I-like receptor-mediated innate immune response. *Proc. Natl. Acad. Sci. U. S. A* **111**, E245-E254 (2014).

236. Xue,J. *et al.* Tumour suppressor TRIM33 targets nuclear beta-catenin degradation. *Nat. Commun.* **6**, 6156 (2015).

237. Kettunen,K.M. *et al.* Trim37-deficient mice recapitulate several features of the multi-organ disorder Mulibrey nanism. *Biol. Open* **5**, 584-595 (2016).

238. Xing,R. *et al.* Spatial memory impairment by TRPC1 depletion is ameliorated by environmental enrichment. *Oncotarget.* **7**, 27855-27873 (2016).

239. Brown,J.E., Krodel,M., Pazos,M., Lai,C., & Prieto,A.L. Cross-phosphorylation, signaling and proliferative functions of the Tyro3 and Axl receptors in Rat2 cells. *PLoS. One.* **7**, e36800 (2012).

240. Cheng,F. *et al.* Inhibition of the NEDD8 conjugation pathway by shRNA to UBA3, the subunit of the NEDD8-activating enzyme, suppresses the growth of melanoma cells. *Asian Pac. J. Cancer Prev.* **13**, 57-62 (2012).

241. Bae,Y., Kho,C.W., Lee,S.Y., Rhim,H., & Kang,S. Hip2 interacts with and destabilizes Smac/DIABLO. *Biochem. Biophys. Res. Commun.* **397**, 718-723 (2010).

242. Zhang,Y. *et al.* Two missense variants in UHRF1BP1 are independently associated with systemic lupus erythematosus in Hong Kong Chinese. *Genes Immun.* **12**, 231-234 (2011).

243. Yuasa-Kawada,J., Kinoshita-Kawada,M., Wu,G., Rao,Y., & Wu,J.Y. Midline crossing and Slit responsiveness of commissural axons require USP33. *Nat Neurosci.* **12**, 1087-1089 (2009).

244. Parsons,J.L. *et al.* USP47 is a deubiquitylating enzyme that regulates base excision repair by controlling steady-state levels of DNA polymerase beta. *Mol. Cell* **41**, 609-615 (2011).

245. Arif,T., Krelin,Y., & Shoshan-Barmatz,V. Reducing VDAC1 expression induces a non-apoptotic role for pro-apoptotic proteins in cancer cell differentiation. *Biochim. Biophys. Acta* **1857**, 1228-1242 (2016).

246. Wang,C.L. *et al.* VPS35 regulates developing mouse hippocampal neuronal morphogenesis by promoting retrograde trafficking of BACE1. *Biol. Open* **1**, 1248-1257 (2012).

247. Wang,L. *et al.* Vps41, a protein involved in lysosomal trafficking, interacts with caspase-8. *Acta Biochim. Pol.* **60**, 37-42 (2013).

248. Moura,D.S. *et al.* Oncogenic Sox2 regulates and cooperates with VRK1 in cell cycle progression and differentiation. *Sci Rep.* **6**, 28532 (2016).

249. Lou,W., Zhang,X., Hu,X.Y., & Hu,A.R. MicroRNA-219-5p Inhibits Morphine-Induced Apoptosis by Targeting Key Cell Cycle Regulator WEE1. *Med. Sci Monit.* **22**, 1872-1879 (2016).

250. Lipowsky,G. *et al.* Exportin 4: a mediator of a novel nuclear export pathway in higher eukaryotes. *EMBO J.* **19**, 4362-4371 (2000).

251. Coppola,M. *et al.* Identification and characterization of YME1L1, a novel paraplegin-related gene. *Genomics* **66**, 48-54 (2000).

252. Hosono,K. *et al.* YPEL5 protein of the YPEL gene family is involved in the cell cycle progression by interacting with two distinct proteins RanBPM and RanBP10. *Genomics* **96**, 102-111 (2010).

253. De,S. & Kline,D. Evidence for the requirement of 14-3-3eta (YWHAH) in meiotic spindle assembly during mouse oocyte maturation. *BMC. Dev. Biol.* **13**, 10 (2013).

254. Kelly,S.M. *et al.* A conserved role for the zinc finger polyadenosine RNA binding protein, ZC3H14, in control of poly(A) tail length. *RNA.* **20**, 681-688 (2014).

255. Ma,J., Mi,C., Wang,K.S., Lee,J.J., & Jin,X. Zinc finger protein 91 (ZFP91) activates HIF-1alpha via NF-kappaB/p65 to promote proliferation and tumorigenesis of colon cancer. *Oncotarget.* **7**, 36551-36562 (2016).

256. Kjaergaard,T., Desdorf,R., Heuck,A., Olsen,A., & Lykke-Hartmann,K. The zinc finger RNA binding protein, ZFR, contributes to axon guidance in Caenorhabditis elegans. *Gene* **557**, 11-18 (2015).

257. Imai,R., Asai,K., Hanai,J., & Takenaka,M. Transgenic mice overexpressing glia maturation factor-beta, an oxidative stress inducible gene, show premature aging due to Zmpste24 down-regulation. *Aging (Albany. NY)* **7**, 486-499 (2015).

258. Gao,S., Hsieh,C.L., Zhou,J., & Shemshedini,L. Zinc Finger 280B regulates sGCalpha1 and p53 in prostate cancer cells. *PLoS. One.* **8**, e78766 (2013).

References for microRNA target genes in prosurvival pathways (axon guidance, ephrin, ERK/MAPK signaling) involved in cell survival, development and cell -cell interactions

259. Wallquist,W. *et al.* Dorsal root ganglion neurons up-regulate the expression of laminin-associated integrins after peripheral but not central axotomy. *J Comp Neurol.* **480**, 162-169 (2004).

260. Madathil,S.K. *et al.* Astrocyte-Specific Overexpression of Insulin-Like Growth Factor-1 Protects Hippocampal Neurons and Reduces Behavioral Deficits following Traumatic Brain Injury in Mice. *PLoS. One.* **8**, e67204 (2013).

261. Zhan,A. *et al.* Decreased expression of Gab2 in patients with temporal lobe epilepsy and pilocarpine-induced rat model. *Synapse* **68**, 168-177 (2014).

262. Marciano,P.G. *et al.* Neuron-specific mRNA complexity responses during hippocampal apoptosis after traumatic brain injury. *J Neurosci* **24**, 2866-2876 (2004).

263. Luksys,G. *et al.* BAIAP2 is related to emotional modulation of human memory strength. *PLoS. One.* **9**, e83707 (2014).

264. Zhuang,B., Su,Y.S., & Sockanathan,S. FARP1 promotes the dendritic growth of spinal motor neuron subtypes through transmembrane Semaphorin6A and PlexinA4 signaling. *Neuron* **61**, 359-372 (2009).

265. MacMillan,K.S. *et al.* Development of proneurogenic, neuroprotective small molecules. *J. Am. Chem. Soc.* **133**, 1428-1437 (2011).

266. Barthet,G. *et al.* Presenilin mediates neuroprotective functions of ephrinB and brain-derived neurotrophic factor and regulates ligand-induced internalization and metabolism of EphB2 and TrkB receptors. *Neurobiol. Aging* **34**, 499-510 (2013).

267. Liu,X., Hawkes,E., Ishimaru,T., Tran,T., & Sretavan,D.W. EphB3: an endogenous mediator of adult axonal plasticity and regrowth after CNS injury. *J Neurosci.* **26**, 3087-3101 (2006).

268. Hwangpo,T.A. *et al.* G Protein-regulated inducer of neurite outgrowth (GRIN) modulates Sprouty protein repression of mitogen-activated protein kinase (MAPK) activation by growth factor stimulation. *J Biol. Chem* **287**, 13674-13685 (2012).

269. Kim,M.S., Kim,J.H., Jin,Y.Z., Kry,D., & Park,B.R. Temporal changes of cFos-like protein expression in medial vestibular nuclei following arsanilate-induced unilateral labyrinthectomy in rats. *Neurosci. Lett.* **319**, 9-12 (2002).

270. Yang,Y. *et al.* EPAC null mutation impairs learning and social interactions via aberrant regulation of miR-124 and Zif268 translation. *Neuron* **73**, 774-788 (2012).

271. Lyeth,B.G. *et al.* Effects of anticholinergic treatment on transient behavioral suppression and physiological responses following concussive brain injury to the rat. *Brain. Res.* **448**, 88-97 (1988).

272. Rahman,A., Khan,K.M., Al-Khaledi,G., Khan,I., & Al-Shemary,T. Over activation of hippocampal serine/threonine protein phosphatases PP1 and PP2A is involved in lead-induced deficits in learning and memory in young rats. *NeuroToxicology* **33**, 370-383 (2012).

273. Dash,P.K., Orsi,S.A., & Moore,A.N. Sequestration of serum response factor in the hippocampus impairs long-term spatial memory. *J Neurochem.* **93**, 269-278 (2005).

274. Guillermin,M.L., Castelletto,M.L., & Hallem,E.A. Differentiation of carbon dioxide-sensing neurons in Caenorhabditis elegans requires the ETS-5 transcription factor. *Genetics* **189**, 1327-1339 (2011).

275. Ning,B., Li,Z., Zhu,N., Hou,G., & Pang,Q. Traumatic brain injury induces a downregulation of MSK1 in rat brain cortex. *J Mol. Neurosci.* **49**, 380-386 (2013).

References for TBI-altered microRNAs implicated in cancer

276. Qin,B. *et al.* MicroRNAs expression in ox-LDL treated HUVECs: MiR-365 modulates apoptosis and Bcl-2 expression. *Biochem. Biophys. Res. Commun.* **410**, 127-133 (2011).

277. Hossain,A., Kuo,M.T., & Saunders,G.F. Mir-17-5p regulates breast cancer cell proliferation by inhibiting translation of AIB1 mRNA. *Mol. Cell Biol.* **26**, 8191-8201 (2006).

278. Calin,G.A. *et al.* Frequent deletions and down-regulation of micro- RNA genes miR15 and miR16 at 13q14 in chronic lymphocytic leukemia. *Proc Natl. Acad. Sci U. S. A* **99**, 15524-15529 (2002).

279. Klein,U. *et al.* The DLEU2/miR-15a/16-1 cluster controls B cell proliferation and its deletion leads to chronic lymphocytic leukemia. *Cancer Cell* **17**, 28-40 (2010).

280. Wang,Y. *et al.* MicroRNAs expression signatures are associated with lineage and survival in acute leukemias. *Blood Cells Mol. Dis.* **44**, 191-197 (2010).

281. Yoshimoto,N. *et al.* Distinct expressions of microRNAs that directly target estrogen receptor alpha in human breast cancer. *Breast Cancer Res. Treat.* **130**, 331-339 (2011).

282. Zhang,X. *et al.* MicroRNA-19 (miR-19) regulates tissue factor expression in breast cancer cells. *J Biol. Chem.* **286**, 1429-1435 (2011).

283. Loven,J. *et al.* MYCN-regulated microRNAs repress estrogen receptor-alpha (ESR1) expression and neuronal differentiation in human neuroblastoma. *Proc Natl. Acad. Sci. U. S. A* **107**, 1553-1558 (2010).

284. Chen,Z.L. *et al.* microRNA-92a promotes lymph node metastasis of human esophageal squamous cell carcinoma via E-cadherin. *J Biol. Chem.* **286**, 10725-10734 (2011).

285. Hurst,D.R. *et al.* Breast cancer metastasis suppressor 1 up-regulates miR-146, which suppresses breast cancer metastasis. *Cancer Res.* **69**, 1279-1283 (2009).

286. Wang,X., Gocek,E., Liu,C.G., & Studzinski,G.P. MicroRNAs181 regulate the expression of p27Kip1 in human myeloid leukemia cells induced to differentiate by 1,25-dihydroxyvitamin D3. *Cell Cycle* **8**, 736-741 (2009).

287. Hashimoto,Y., Akiyama,Y., Otsubo,T., Shimada,S., & Yuasa,Y. Involvement of epigenetically silenced microRNA-181c in gastric carcinogenesis. *Carcinogenesis* **31**, 777-784 (2010).
